# A revision of dragon millipedes IV: the new genus *Spinaxytes*, with the description of nine new species (Diplopoda, Polydesmida, Paradoxosomatidae)

**DOI:** 10.3897/zookeys.797.29510

**Published:** 2018-11-19

**Authors:** Ruttapon Srisonchai, Henrik Enghoff, Natdanai Likhitrakarn, Somsak Panha

**Affiliations:** 1 Biological Sciences Program, Faculty of Science, Chulalongkorn University, Phaya Thai Road, Patumwan, Bangkok 10330, Thailand; 2 Animal Systematics Research Unit, Department of Biology, Faculty of Science, Chulalongkorn University, Phayathai Road, Patumwan, Bangkok 10330, Thailand; 3 Natural History Museum of Denmark, University of Copenhagen, Universitetsparken 15, DK-2100 København Ø, Denmark; 4 Division of Plant Protection, Faculty of Agricultural Production, Maejo University, San Sai, Chiang Mai 50290, Thailand

**Keywords:** dragon millipede, endemic, new species, taxonomy, Thailand

## Abstract

Nine new species constituting the ‘*spiny*’ group of dragon millipedes are assigned to the new genus *Spinaxytes* Srisonchai, Enghoff & Panha, **gen. n.** Seven new species are described from Thailand: *S.biloba* Srisonchai, Enghoff & Panha, **sp. n.** and *S.palmata* Srisonchai, Enghoff & Panha, **sp. n.** from Surat Thani Province, *S.hasta* Srisonchai, Enghoff & Panha, **sp. n.** from Chumphon Province, *S.krabiensis* Srisonchai, Enghoff & Panha, **sp. n.** (type species) and *S.sutchariti* Srisonchai, Enghoff & Panha, **sp. n.** from Krabi Province, *S.uncus* Srisonchai, Enghoff & Panha, **sp. n.**, and *S.macaca* Srisonchai, Enghoff & Panha, **sp. n.** from Phang Nga Province; as well as one from Malaysia, *S.tortioverpa* Srisonchai, Enghoff & Panha, **sp. n.**, and one from Myanmar, *S.efefi* Srisonchai, Enghoff & Panha, **sp. n.** The new genus is endemic to South Myanmar, South Thailand, and Malaysia, and all new species are restricted to limestone habitats. All were exclusively found living on humid rock walls and/or inside small caves. Complete illustrations of external morphological characters, an identification key, and a distribution map are provided.

## Introduction

This is the fourth paper in a series of articles about revision of the dragon millipedes. Srisonchai et al. (2018a) provided general information on dragon millipedes, split *Desmoxytes* Chamberlin, 1923, sensu Golovatch and Enghoff (1994) into five genera based on morphological and genetic data, and revised the genus *Desmoxytes* in its new, restricted sense. Subsequently, Srisonchai et al. (2018a, b) described two new genera of dragon millipedes containing several new species and several species transferred from *Desmoxytes*. In the present study, we describe nine new species constituting the group that we (Srisonchai et al. 2018a) provisionally named the ‘*spiny*’ group, of which no species has hitherto been named, and assign them to *Spinaxytes* gen. n.

The new genus is narrowly distributed in the Malay Peninsula (Malaysia, Myanmar, and Thailand).

## Materials and methods

### Specimen collection and preservation

Specimens were hand-collected from many localities throughout South Thailand, in some parts of Malaysia and in southern Myanmar. We also observed the habitats of all specimens. Specimens were mostly stored in 70% ethanol for morphological study and partly in 95% ethanol for molecular analysis. Latitude, longitude, and elevation were recorded by using a Garmin GPSMAP 60 CSx, and all coordinates and elevations were checked with Google Earth.

The main collectors in this work were staff and students of the Animal Systematics Research Unit, Department of Biology, Faculty of Science, Chulalongkorn University which we here refer to as ‘ASRU members’.

### Illustrations

All living specimen photos were taken with a Nikon D700 equipped with a AF-S VR Micro-Nikkor 105 mm lens during fieldwork. Newly collected specimens preserved in ethanol were imaged with an Olympus DP72 camera on an Olympus SZX16 stereomicroscope, using image stacking Cell-D auto-montage software. Scanning electron micrographs were generated with a JEOL – JSM–5410 LV. All samples studied with SEM were carefully dissected under a microscope, mounted on aluminium stubs, and coated with gold. After imaging with SEM, all objects were removed and kept in dry condition. Drawings were outlined under a stereo microscope (Leica Wild M10) with a drawing tube and finished using dot-line technique (stipple). Plates were composed in Adobe Photoshop CS6.

### Morphological descriptions

We use morphological terminology according to previous taxonomic publications (Chamberlin 1923; Jeekel 1964, 1980, 2003; Golovatch and Enghoff 1994; Enghoff et al. 2007; Golovatch et al. 2012; Srisonchai et al. 2016, 2018a, 2018b, 2018c. Details of gonopodal terms are shown in the gonopod terminology section below.

### Gonopod terms for the genus *Spinaxytes* gen. n., and their abbreviations

## Taxonomy

### Class Diplopoda Blainville in Gervais, 1844

#### Order Polydesmida Pocock, 1887

##### Family Paradoxosomatidae Daday, 1889

###### Subfamily Paradoxosomatinae Daday, 1889

####### Tribe Orthomorphini Brölemann, 1916

######## Spinaxytes

Taxon classificationAnimaliaPolydesmidaParadoxosomatidae

Srisonchai, Enghoff & Panha gen. n.

http://zoobank.org/EB550BAF-CFF4-4683-9E00-D00C17227870

######### Type species.

*Spinaxyteskrabiensis* Srisonchai, Enghoff & Panha, gen. et sp. n.

######### Diagnosis.

The genus *Spinaxytes* gen. n. is characterized by:

1. Paraterga spiniform.

2. Metaterga with two rows of tubercles/cones/spines.

3. Postfemoral part of gonopod conspicuous, demarcated from femur by deep mesal and lateral sulci.

4. Lamina lateralis distinctly demarcated from lamina medialis.

5. Lamina medialis very long, curved, larger and longer than lamina lateralis.

######### Etymology.

The name is a noun in apposition; from the Latin ‘*spina*’, referring to the spine-like paraterga of all constituent species; ‘–*xytes*’ ensures harmony with *Desmoxytes* (and its synonym ‘*Pteroxytes*’).

######### Included species.

1. *Spinaxytesbiloba* Srisonchai, Enghoff & Panha, sp. n.

2. *Spinaxytesefefi* Srisonchai, Enghoff & Panha, sp. n.

3. *Spinaxyteshasta* Srisonchai, Enghoff & Panha, sp. n.

4. *Spinaxyteskrabiensis* Srisonchai, Enghoff & Panha, sp. n.

5. *Spinaxytesmacaca* Srisonchai, Enghoff & Panha, sp. n.

6. *Spinaxytespalmata* Srisonchai, Enghoff & Panha, sp. n.

7. *Spinaxytessutchariti* Srisonchai, Enghoff & Panha, sp. n.

8. *Spinaxytestortioverpa* Srisonchai, Enghoff & Panha, sp. n.

9. *Spinaxytesuncus* Srisonchai, Enghoff & Panha, sp. n.

######### Remarks.

The new genus is easily distinguished from other genera of dragon millipedes by having spiniform paraterga, lamina lateralis smaller and shorter than lamina medialis, lamina medialis long and curved. Some species of the genus *Hylomus* Cook & Loomis, 1924, share spine-like paraterga; however, the gonopod details are totally different.

**Figure 1.**
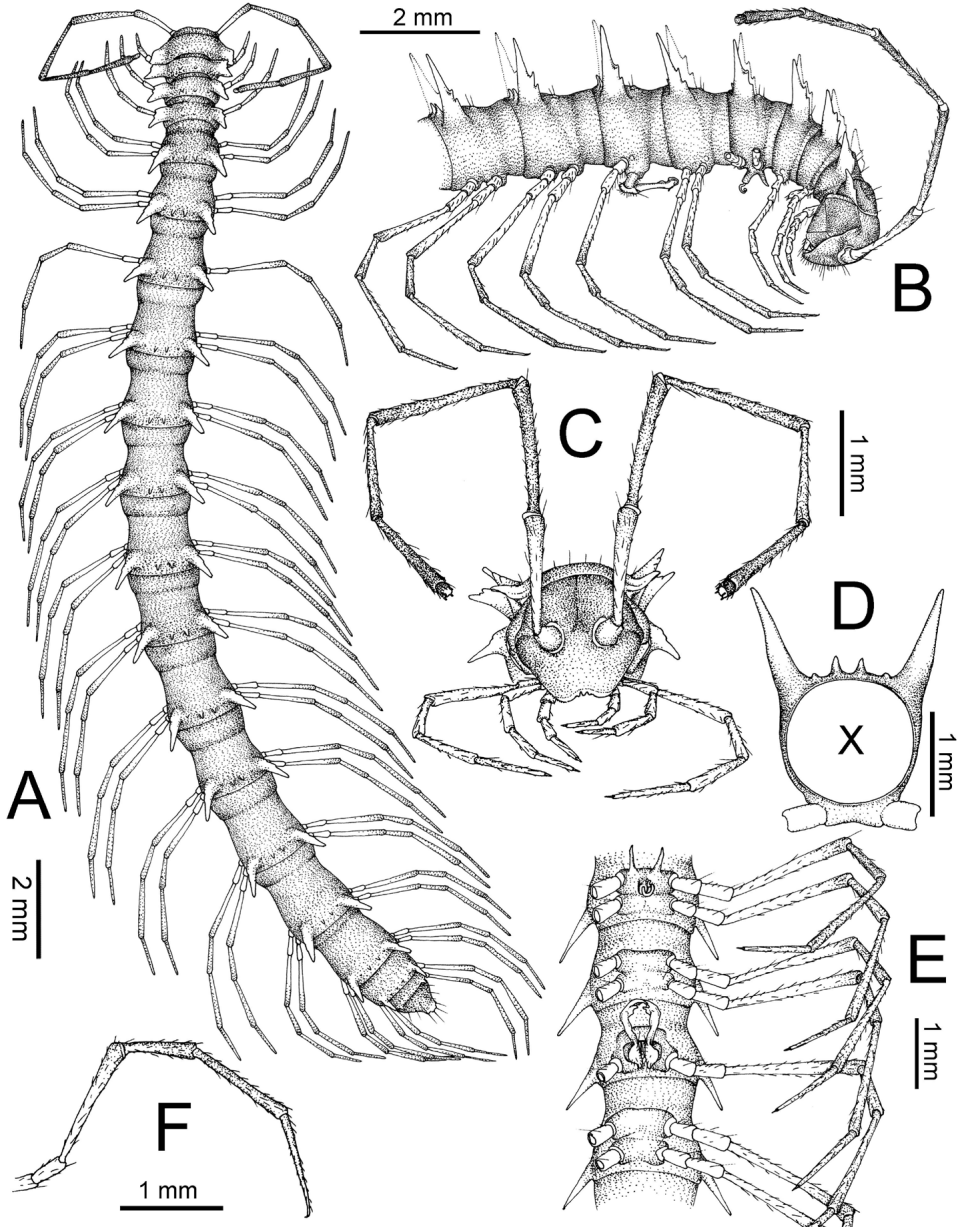
General body characters of *Spinaxytes* gen. n. (*S.tortioverpa* sp. n., ♂ paratype, CUMZ-pxDGT00220) **A** whole body **B** anterior body part **C** head and antennae **D** midbody ring **E** body rings 5–8, showing gonopods and sternal lobe between coxae 4 **F** leg 13.

**Figure 2.**
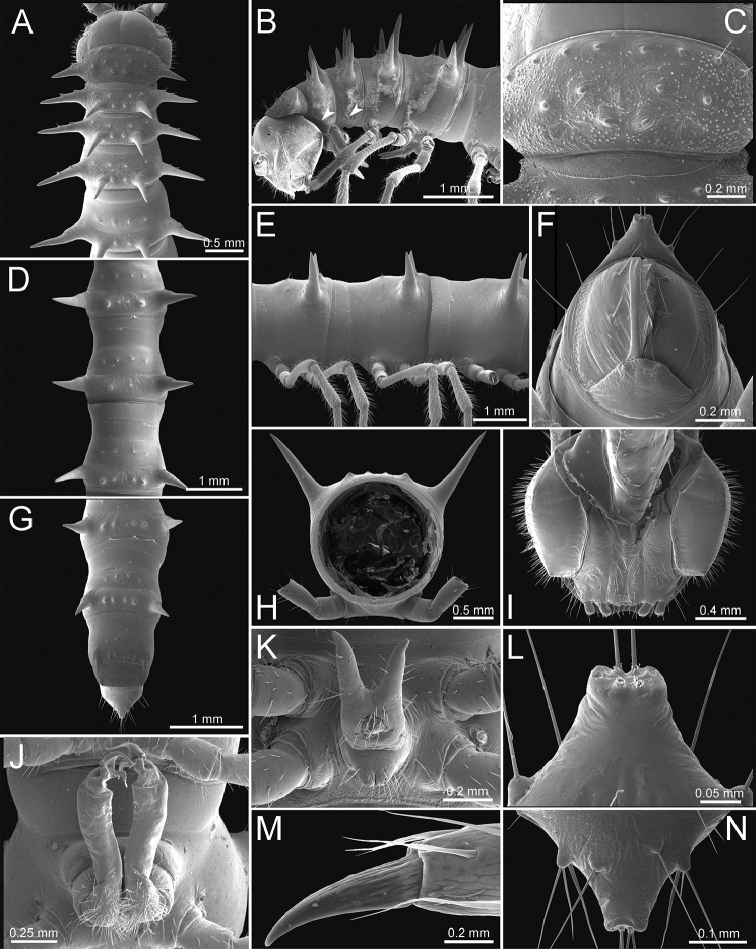
General body characters of *Spinaxytes* gen. n. (*S.palmata* sp. n., ♂ paratype, CUMZ-pxDGT00216) – SEM images **A, B** anterior body part (arrowheads point to pleurosternal carinae) **C** collum **D, E** body rings 9–11 **F** telson **G** posteriormost rings and telson **H** body ring 10 **I** mouth parts, ventral view **J** gonopods **K** sternal lobe between coxae 4 **L, N** tip of epiproct **M** tip of tarsus and claw of leg 13.

**Figure 3.**
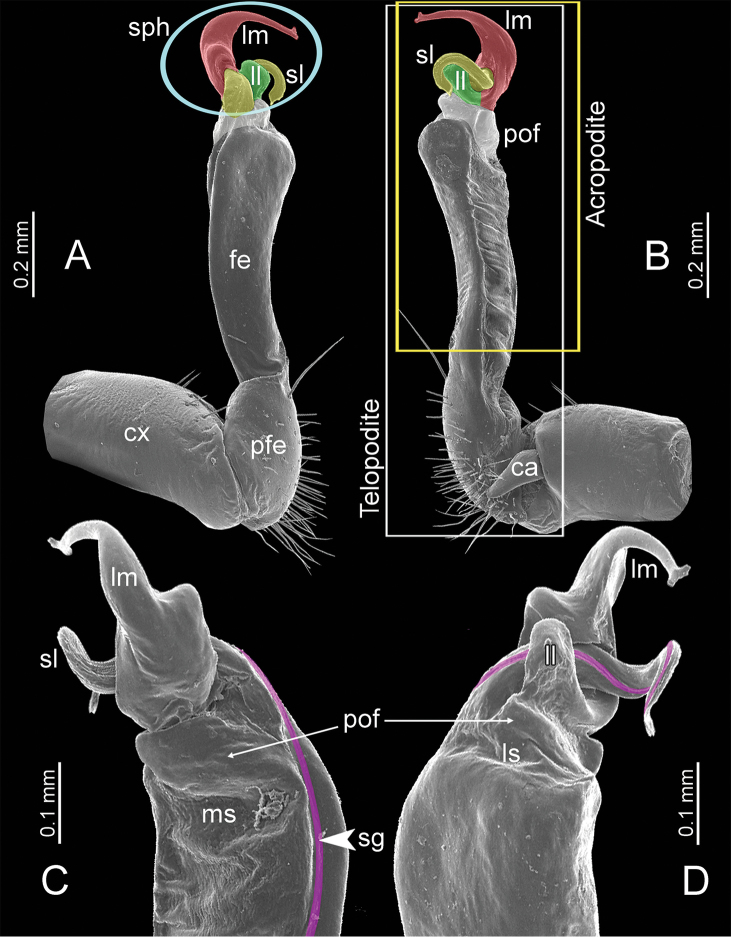
SEM images of right gonopod of *Spinaxytes* gen. n. (*Spinaxyteskrabiensis* sp. n., ♂ paratype, CUMZ-pxDGT00212) **A** lateral view **B** mesal view **C** dorsal view **D** ventral view. Key: red = lamina medialis (lm), yellow = solenomere (sl), green = lamina lateralis (ll), purple = seminal groove (sg).

**Figure 4.**
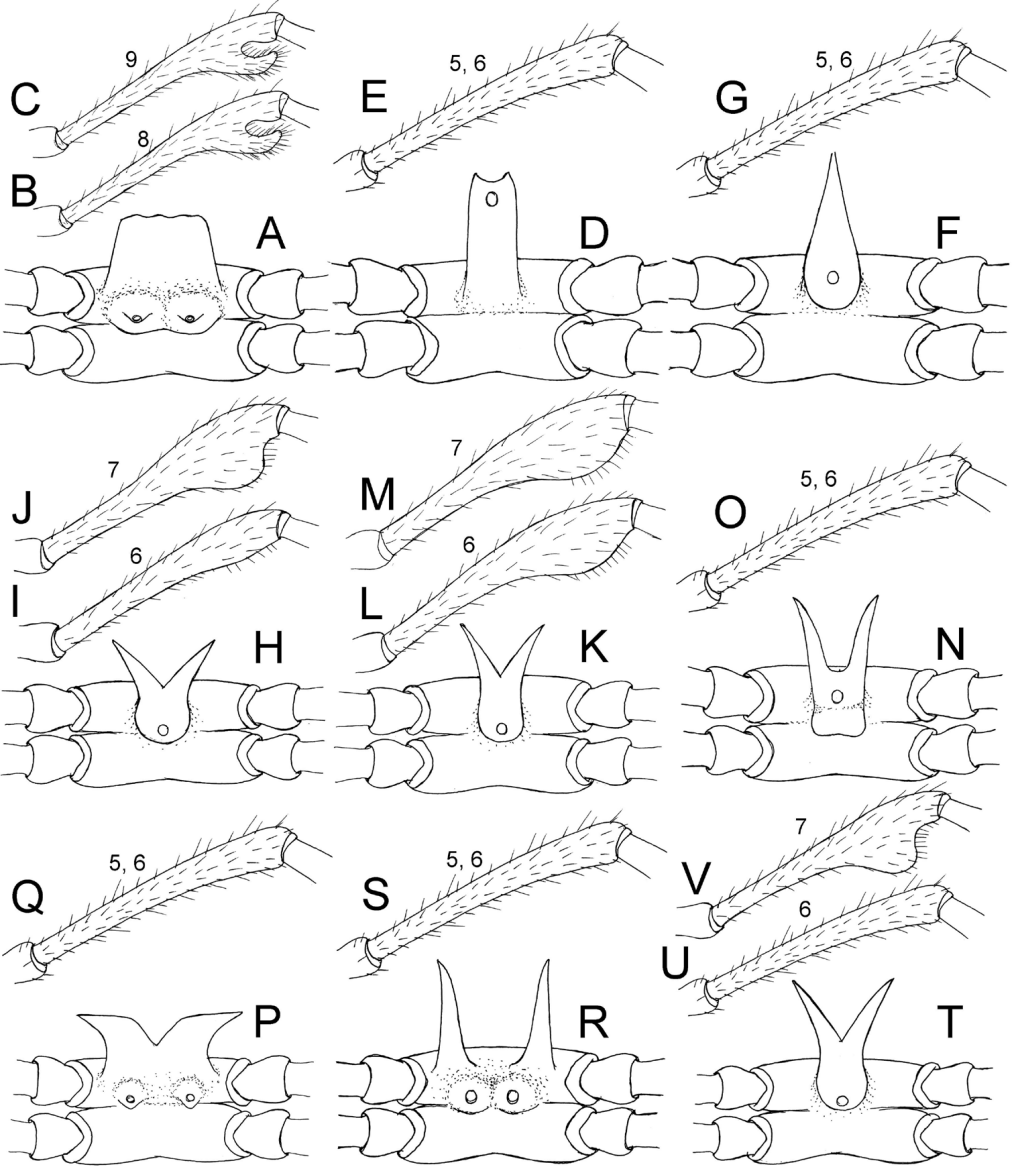
Male femora and sternal lobe between male coxae 4 of *Spinaxytes* gen. n. **A–C***S.biloba* sp. n. (**A** Sternal lobe **B** Femur 8 **C** Femur 9) **D, E***S.efefi* sp. n. (**D** Sternal lobe **E** Femur 5 or 6) **F, G***S.hasta* sp. n. (**F** Sternal lobe **G** Femur 5 or 6) **H–J***S.krabiensis* sp. n. (**H** Sternal lobe **I** Femur 6 **J** Femur 7) **K–M***S.macaca* sp. n. (**K** Sternal lobe **L** Femur 6 **M** Femur 7) **N, O***S.palmata* sp. n. (**N** Sternal lobe **O** Femur 5 or 6) **P, Q***S.sutchariti* sp. n. (**P** Sternal lobe **Q** Femur 5 or 6) **R, S***S.tortioverpa* sp. n. (**R** Sternal lobe **S** Femur 5 or 6) **T–V***S.uncus* sp. n. (**T** Sternal lobe **U** Femur 6 **V** Femur 7).

**Figure 5.**
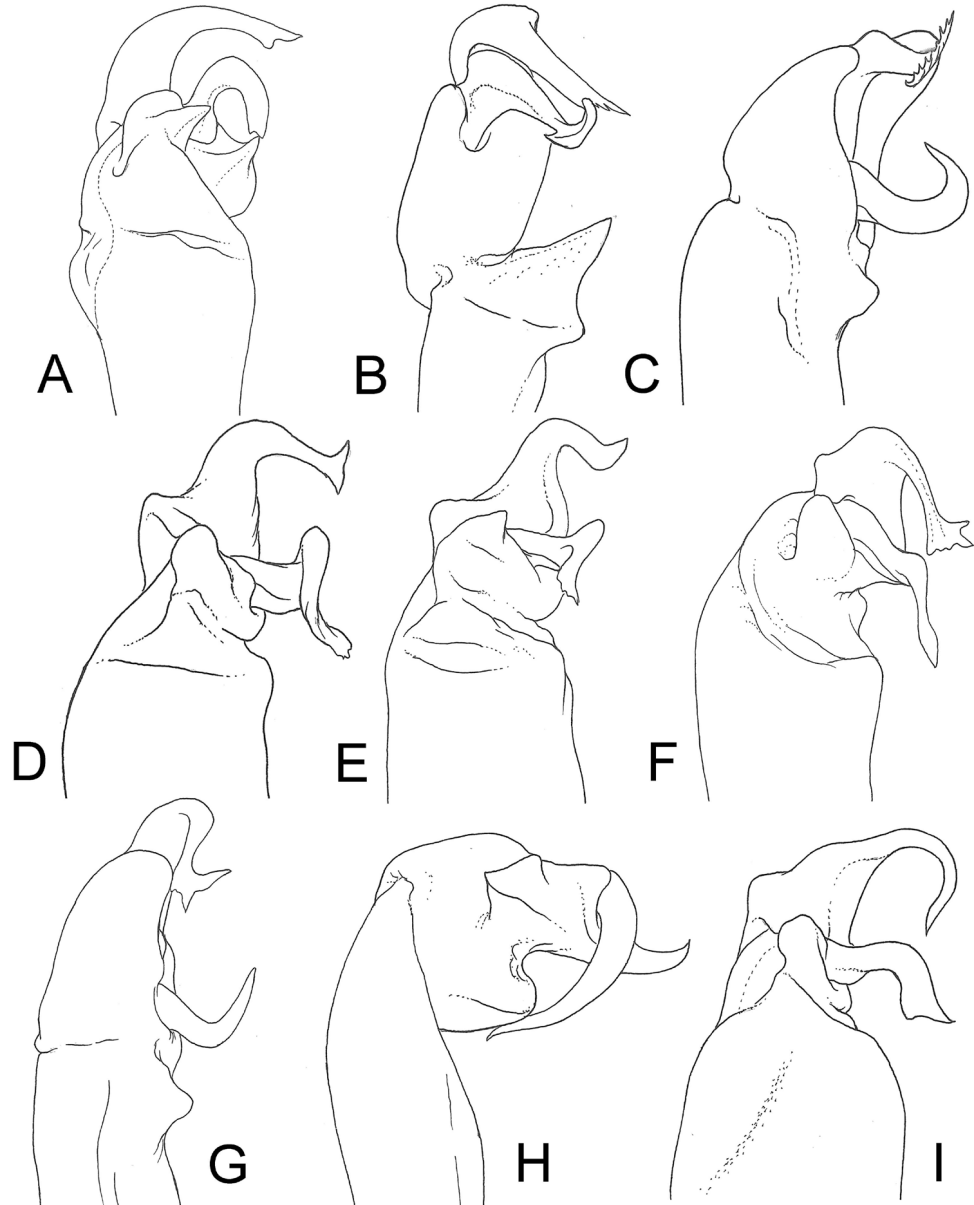
Right gonopods of *Spinaxytes* gen. n. (ventral view) **A***S.biloba* sp. n. **B***S.efefi* sp. n. **C***S.hasta* sp. n. **D***S.krabiensis* p. n. **E***S.macaca* sp. n. **F***S.palmata* sp. n. **G***S.sutchariti* sp. n. **H***S.tortioverpa* sp. n. **I***S.uncus* sp. n.

**General description of the genus *Spinaxytes*.** The description applies to adult males and females, except for the gonopods and when “male” is specified (Figs 1, 2, 4). The general description of the gonopods is based mainly on *Spinaxyteskrabiensis* gen. et sp. n. (Figs 3, 5).

***Size***. Body length ca. 18–33 mm (male) ca. 16–33 mm (female), width 1.0–2.2 mm (male) 1.3–2.9 mm (female), size varies between species, usually female a bit longer than male.

***Colour***. Most species in life with dark brown colour. Colour in alcohol: all specimens partly faded to pale brown after 5 years’ preservation in alcohol; specimens kept in darkness faded more slowly.

***Antennae*** (Figure 1A–C). Extremely long and slender, covered by delicate setation, usually reaching backwards to body rings 7–10 (male) and 6–8 (female) when stretched dorsally. Antennomere 3 = 4 > 5 ≥ 2 > 6 > 1 > 7 > 8.

***Head***. Delicately setose; vertex, labrum and genae sparsely setose; epicranial suture conspicuous as a deep, brown or black stripe.

***Collum*** (Figure 2A, C). With three regular transverse rows of setiferous tubercles/cones; number of tubercles/cones in each row varies between species. Paraterga wing-like/spiniform, usually elevated at ca. 10°–30°, directed laterad/caudolaterad/caudad, with one or two conspicuous/inconspicuous notches at lateral margin.

***Tegument.*** Quite dull, sometimes shining; collum, metaterga and surface below paraterga smooth/microgranulate; prozona finely shagreened; paraterga, epiproct and sterna smooth. Stricture between prozona and metazona shallow, wide.

***Metaterga*** (Figure 2A, D, G). With two regular transverse rows of setiferous cones/tubercles (in anterior row) and cones/spines (in posterior row); number of tubercles/cones/spines in each row varies between species. Transverse sulcus on metaterga shallow and wide in body rings 5–18. Mid-dorsal (axial) line missing.

***Pleurosternal carinae*** (Figure 2B). Forming a complete, tooth-like crest on ring 2, a short ridge on ring 3, missing on remaining body rings.

***Paraterga*** (Figs 1A, B, D; 2A, B, D, E, G, H). Spiniform, long (except *S.biloba* sp. n.: quite short), extremely elevated at ca. 45°–80° (male) 40°–70° (female). Callus and shoulder poorly developed, inconspicuous. Anterior margin with two distinct denticles; on body rings 9, 10, 12, 13, 15–19 without a third denticle at lateral margin near tip. Degree of elevation of paraterga in male usually higher than in female. Posterior angle straight. Tip pointed and sharp. Ozopore visible from dorsal/dorsolateral/lateral view, round, small.

***Telson*** (Figure 2F, G, L, N). Epiproct usually long, apically with two pairs of conspicuous setae (spinnerets) arranged at the corners of a square, not in a depression, anterior pair close to apical tubercles. Paraprocts convex. Hypoproct usually subtrapeziform, sometimes subsemicircular/subtriangular; caudal margin often round, sometimes subtruncate/angular, with two conspicuous/ inconspicuous setiferous tubercles.

***Sterna*** (Figs 1E, 2K). Sparsely setose, cross-impressions shallow in all species. Sternal lobe between male coxae 4 varies in shape; subtrapeziform/long subrectangular/bifurcate/spear-like; one or two pores seen in posterior view.

***Legs*** (Figs 1F, 2M). Extremely long and slender. Relative length of podomeres: femur ≥ tibia > tarsus ≥ postfemur > prefemur > coxa > claw. Male femora mostly without modification, sometimes male femora 6, 7 or 7 or 8, 9 with hump/apophysis ventrally in distal part.

***Gonopods*** (Figure 3). Coxa shorter than femur, sometimes subequal in length to femur. Cannula long and slender. Telopodite erect. Prefemoral part usually almost half as long, sometimes ca. 2/3 as long as femur. Acropodite erect. Femur long and straight. Seminal groove running entirely on mesal surface of femur. Mesal sulcus and lateral sulcus conspicuous, deep. Postfemoral part conspicuous, usually small and narrow, sometimes broad and wide, rarely very large. Solenophore variously modified in shape between species: lamina lateralis obviously demarcated from lamina medialis, smaller and shorter than lamina medialis; lamina medialis long, base stout, slightly attenuated near the curved tip. Solenomere long, slender, curved, supported by solenophore.

######### Distribution and habitat.

All species of the new genus are allopatric. However, some of the new species can be found in syntopy with some species of *Desmoxytes*. The nine species described here are confined to limestone habitats and have narrow distributions; we therefore regard them as endemic. All species blend perfectly with habitat environment, mostly living on humid rock walls and/or inside small caves.

According to the current knowledge, *Spinaxytes* gen. n. is distributed only in the Malay Peninsula (south Myanmar, south Thailand, and north Malaysia): Myanmar: Thanintharyi Region; Thailand: Chumphon Province (Thung Tako, Mueng Chumphon, Lang Suan and Sawi Districts), Surat Thani Province (Phanom District), Phang Nga Province (Mueng Phang Nga and Takua Thung Districts), Krabi Province (Ao Luek and Muaeng Krabi Districts); Malaysia: Perak State (Figure 30).

######## Key to species of *Spinaxytes* gen. n. (based mainly on males)

**Table:** 

1	Male femora 6–9 without modification (e.g., Figs 4E, G; 10J; 13J)	**2**
–	Male femora 6 and 7, or 7, or 8 and 9 humped/with apophyses (e.g., Figs 4B, C, L, M; 7J; 15J)	**6**
2	Collum with 5+5 tubercles in anterior row, 2+2 tubercles in intermediate row, 3+3 tubercles in posterior row (Figure 10A). Metaterga 2–8 with 3+3 cones in anterior row and 3+3 cones in posterior row; metaterga 9–19 with 3+3 cones in anterior row and 4+4 cones in posterior row (Fig. 2A, C, E)	***S.efefi* sp. n.**
–	Collum with 4+4 tubercles/cones in anterior row, 1+1 in intermediate row, 2+2 in posterior row (e.g., Figs 16A, 28A). Metaterga 2–19 with 2+2 tubercles/cones in anterior row and 2+2/3+3 tubercles/cones/spines in posterior row (e.g., Figs 16A, C, E; 28A, C, E)	**3**
3	Sternal lobe between male coxae 4 bilobed/bifurcate/subtrapeziform (e.g., Figs 4A, K, P, R; 7F, G; 19F, G)	**4**
–	Sternal lobe between male coxae 4 spear-like (not bilobed, not bifurcate, not subtrapeziform) (Figs 4F; 13F, G)	***S.hasta* sp. n.**
4	Postfemoral part very large, angled 90 degrees with femoral part (Figs 5H, 26C–F). Lamina lateralis divided into two lobes; first lobe spine-like, long; second lobe smaller, ridge-like (Figs 5H; 26A, C, D). Lamina medialis curving up (Figs 5H; 26C, E). Solenomere longer than lamina medialis (Figs 5H; 26A–D)	***S.tortioverpa* sp. n.**
–	Postfemoral part small, not angled 90 degrees with femoral part (e.g., Figs 5F, G; 23C; 25C). Lamina lateralis not divided into two lobes (e.g., Figs 5F, G; 23C; 25C, F). Lamina medialis curving down (e.g., Figs 5F, G; 23C; 25C, D). Solenomere approximately equal in length to lamina medialis (e.g., Figs 5F, G; 23C; 25C)	**5**
5	Lamina lateralis small (Figs 5F, 23C). Solenomere circular in transverse section, curving down (Fig. 23C, E)	***S.palmata* sp. n.**
–	Lamina lateralis large (Figs 5G; 25C, F). Solenomere flat in transverse section, curving up (Fig. 25C–E)	***S.sutchariti* sp. n.**
6	Only male femora 7 modified (Figs 4V, 28J)	***S.uncus* sp. n.**
–	Male femora 6 and 7, or 8 and 9 modified (e.g. Figs 4B, C, L, M; 7J; 19J)	**7**
7	Lamina medialis with process-like lobe at base (Fig. 8D). Sternal lobe between male coxae 4 subtrapeziform (Figs 4A; 7F, G). Male femora 8 and 9 with apophyses	***S.biloba* sp. n.**
–	Lamina medialis without process-like lobe at base (Figs 17D, E; 20D, E). Sternal lobe between male coxae 4 incompletely bilobed, fork-like (Figs 4H, K; 16F, G; 19F, G). Male femora 6 and 7 modified as humped ventrally in distal portion	**8**
8	Paraterga extremely long (Fig. 16D). Lamina lateralis distally round (Fig. 17C). Tip of lamina lateralis terminating in two lobes (Figs 3C, D; 17C, D, E)	***S.krabiensis* sp. n.**
–	Paraterga moderately long (Fig. 19D). Lamina lateralis distally protruding, lobe-like (Fig. 20C). Tip of lamina lateralis bent, terminating in one lobe (Fig. 20C, D, E)	***S.macaca* sp. n.**

######## Species descriptions

######### Spinaxytes biloba

Taxon classificationAnimaliaPolydesmidaParadoxosomatidae

Srisonchai, Enghoff & Panha sp. n.

http://zoobank.org/DAC03327-012B-4096-846C-468C40558DDE

**Figure 6.**
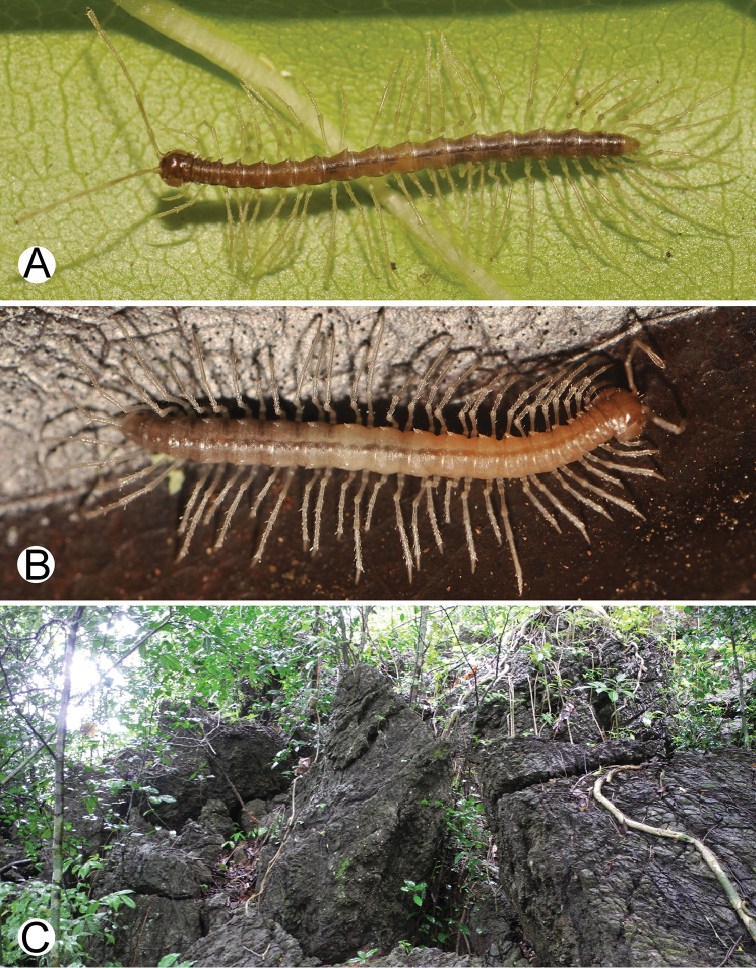
Photographs of live *Spinaxytesbiloba* sp. n. and habitat **A** ♂ paratype, CUMZ-pxDGT00206 **B** ♀ paratype, CUMZ-pxDGT00206 **C** Habitat.

**Figure 7.**
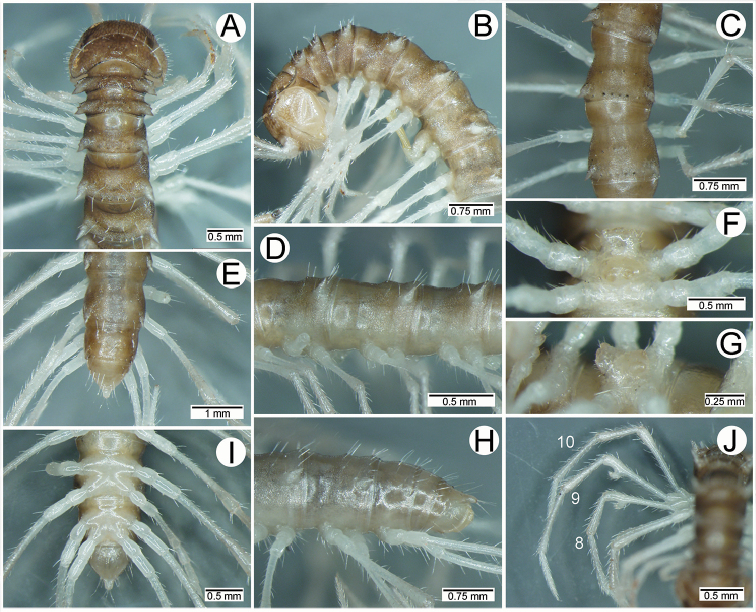
*Spinaxytesbiloba* sp. n., ♂ paratype, CUMZ-pxDGT00206 **A, B** anterior body part **C, D** body rings 8–10 **E, H, I** posteriormost body rings and telson **F, G** sternal lobe between coxae 4 **J** legs 6–10.

**Figure 8.**
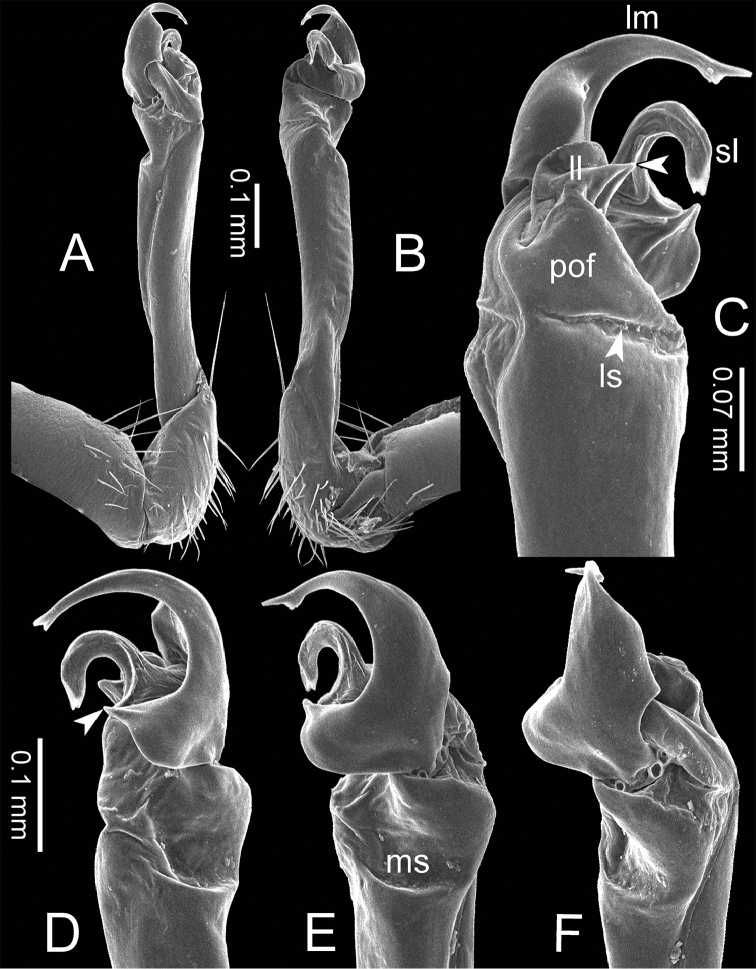
*Spinaxytesbiloba* sp. n., paratype, CUMZ-pxDGT00206 – right gonopod **A** lateral view **B** mesal view **C** ventral view (unlabelled arrowhead points to lobe on lamina lateralis) **D** mesodorsal view (arrowhead points to lobe at base of lamina medialis) **E** dorsal view **F** laterodorsal view.

Figs 4A–C , 5A , 6 , 7 , 8

########## Material examined.

**Holotype.** ♂, THAILAND, Surat Thani Province, Phanom District, near Khlong Phanom National Park, Pha Daeng, 8°53'41"N, 98°33'12"E, ca. 67 m a.s.l., 7 Aug. 2016, ASRU members leg. (CUMZ-pxDGT00205). **Paratypes.** 17 ♂♂, 24 ♀♀, same data as for holotype (CUMZ- pxDGT00206); 1 ♂, 1 ♀, same data as for holotype (ZMUC00040249); 1 ♂, 1 ♀, same data as for holotype (NHMW9423). **Further specimens, not paratypes.** 5 ♂♂, 3 ♀♀, THAILAND, Surat Thani Province, Phanom District, near Khlong Phanom National Park, Pha Daeng, 8°53'41"N, 98°33'12"E, ca. 67 m a.s.l., 6 Aug. 2015, ASRU members leg. (CUMZ).

########## Etymology.

The species name is an adjective, refers to the two additional process-like lobes on the solenophore (one on lamina lateralis and one on lamina medialis).

########## Diagnosis.

Differs from other species by having: metaterga 5–19 with 2+2 cones in anterior row and 3+3 cones in posterior row; sternal lobe between male coxae 4 subtrapeziform; male femora 8 and 9 with apophyses distally; lamina lateralis with an additional process-like protruding lobe; lamina medialis basally with an additional protruding process-like lobe.

########## Description.

SIZE. Length 15–17 mm (male), 16–18 mm (female); width of midbody metazona 1.0–1.2 mm (male), 1.3–1.5 mm (female). Width of rings 2 = 3 < 4 < collum < 5 < head = 6–17, thereafter body gradually tapering towards telson.

***Colour*** (Figure 6A, B). Specimens in life brown/pale brown; paraterga brownish white; head, antennae (except whitish distal part of antennomeres 7 and 8) and collum brown; prozona, metaterga (except white spines in posterior row) and surface below paraterga brown/pale brown; sterna pale brown/whitish brown; epiproct and legs whitish brown; a few basal podomeres whitish brown/white.

***Antennae***. Reaching to body ring 7 or 8 (male) and 6 (female) when stretched dorsally.

***Collum*** (Figure 7A). With three transverse rows of setiferous cones, 4+4 in anterior row, 1+1 in intermediate row and 2+2 in posterior row; with one inconspicuous setiferous notch at lateral margin; paraterga wing-like, quite short, tip blunt, elevated at ca. 15°–20° (male) 10°–20° (female), directed almost caudad.

***Tegument***. Quite shining; collum coarsely microgranulate; metaterga and surface below paraterga finely microgranulate.

***Metaterga*** (Figure 7A, C, E). With two transverse rows of setiferous cones; metaterga 2–4 with 2+2 cones in anterior row and 2(3)+2(3) cones in posterior row; metaterga 5–19 with 2+2 cones in anterior row and 3+3 cones in posterior row; all cones subequal in length and size. An additional cone-like denticle at base of paraterga near anterior row of cones.

***Paraterga*** (Figure 7A–D, H). Quite short; directed dorsocaudad on body rings 3–17, elevated at ca. 60°–70° (male) 55°–70° (female), directed more caudad on body ring II and increasingly so on rings 18 and 19. Denticle of paraterga located at base of paraterga and very close to anterior row of cones on metaterga. Ozopore visible in lateral view.

***Telson*** (Figure 7E, I, H). Epiproct quite long; tip subemarginate; lateral setiferous tubercles conspicuous; apical tubercles conspicuous. Hypoproct subtrapeziform, wide; caudal margin round (in some specimens subtruncate), with inconspicuous setiferous tubercles.

***Sterna*** (Figs 4A; 7F, G). Sternal lobe between male coxae 4 subtrapeziform, broad, and thin, tips subtruncate, in situ directed ventroanteriad; posterior surface of sternal lobe with two pores borne on swollen and short lobe.

***Legs*** (Figs 4B, C; 7J). Male femora 8 and 9 with apophyses distally.

***Gonopods*** (Figs 5A, 8). Coxa shorter than femur. Prefemoral part ca. half as long as femur. Femur not enlarged distally. Postfemoral part broad. Mesal sulcus wide; lateral sulcus narrow. Solenophore subequal in size to postfemoral part: lamina lateralis small and short; with a protruding lobe, process-like, directed mesad; apically round: lamina medialis long; base enlarged and stout, slightly attenuated near the tip, basally with a protruding lobe, process-like, directed mesad; tip curving down, bifurcating into two small spines. Solenomere curving down, compressed in transverse section, tip directed posteriad.

########## Distribution and habitat

(Figure 6C). Known only from the type locality which is a small isolated limestone mountain between Khao Sok and Khlong Phanom National Parks. The new species blended perfectly with the humid rock walls, and most specimens were found inside rock holes/crevices. *S.biloba* sp. n. co-occurs with *Desmoxytescervina* (Pocock, 1895) (Srisonchai et al. 2018a) in the same habitat. Several attempts (2017–2018) have been made to find further specimens near the type locality, but none were found. As the new species has only been found at the type locality only, we regard *S.biloba* sp. n. as endemic to Thailand.

########## Remarks.

Among all *Spinaxytes* species, *S.biloba* sp. n. is obviously the smallest (length 15–18 mm, width of midbody metazona 1.0–1.5 mm), and the live pale brown colouration is lighter than that of other species.

######### Spinaxytes efefi

Taxon classificationAnimaliaPolydesmidaParadoxosomatidae

Srisonchai, Enghoff & Panha sp. n.

http://zoobank.org/935306A6-160D-4903-B3B4-BE046F963661

**Figure 9.**
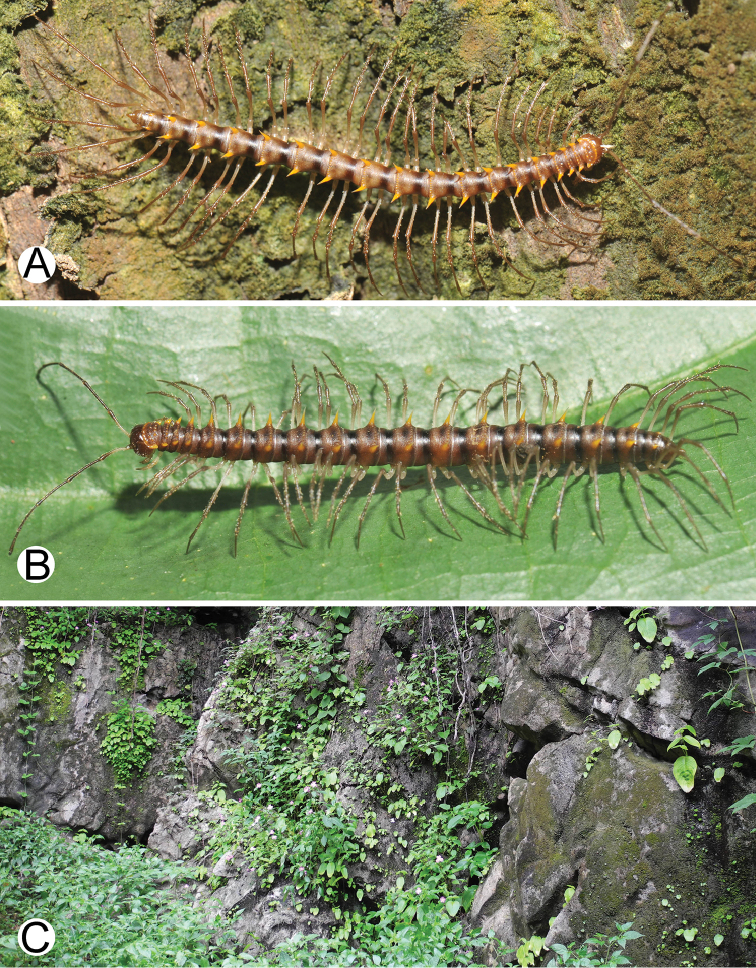
Photographs of live *Spinaxytesefefi* sp. n. and habitat **A** ♂ paratype, CUMZ-pxDGT00208 **B** ♀ paratype, CUMZ-pxDGT00208 **C** habitat.

**Figure 10.**
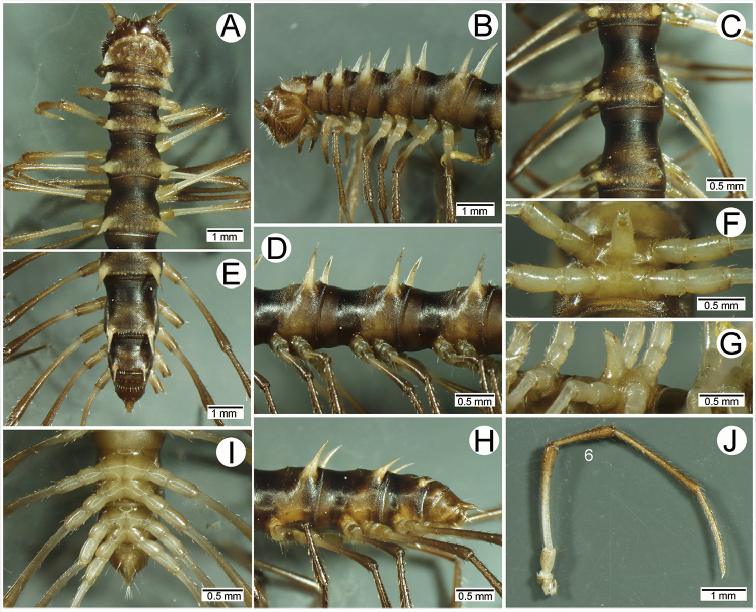
*Spinaxytesefefi* sp. n., ♂ paratype, CUMZ-pxDGT00208 **A, B** anterior body part **C, D** body rings 9–11 **E, H, I** posteriormost body rings and telson **F, G** sternal lobe between coxae 4 **J** leg 6.

**Figure 11.**
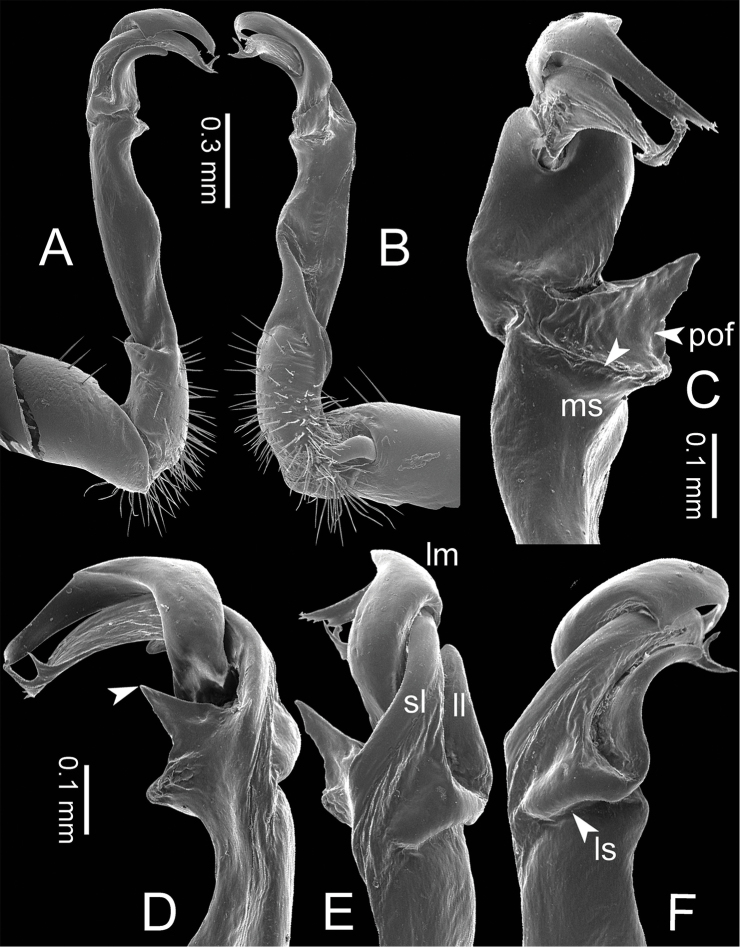
*Spinaxytesefefi* sp. n., paratype, CUMZ-pxDGT00208 – right gonopod **A** lateral view **B** mesal view **C** ventral view (unlabelled arrowhead points to a ridge on postfemoral part) **D** mesodorsal view (arrowhead points to a triangular process on postfemoral part) **E** dorsal view **F** laterodorsal view.

Figs 4D, E ; 5B ; 9 , 10 , 11

########## Material examined.

**Holotype.** ♂, MYANMAR, Tanintharyi Region, Myeik, 20 km northeast of Monoron, Lenya National Park, limestone mountain near Ngawun Chaung River, 11°40'20"N, 99°13'30"E, ca. 64 m a.s.l., 9 Jun. 2015, FFI staff and ASRU members leg. (CUMZ-pxDGT00207). **Paratypes.** 20 ♂♂, 25 ♀♀, same data as for holotype (CUMZ-pxDGT00208); 1 ♂, 1 ♀, same data as for holotype (ZMUC00040250); 1 ♂, 1 ♀, same data as for holotype (ZMUM); 1 ♂, 1 ♀, same data as for holotype (NHMW9422); 1 ♂, 1 ♀, same data as for holotype (NHMUK).

########## Etymology.

The name is an artificially constructed homophone (*efefi* = FFI) honouring FFI (Fauna and Flora International, Myanmar), an organization for biodiversity conservation; in recognition of their hard work to protect wildlife including invertebrates.

########## Diagnosis.

Sternal lobe between male coxae 4 not bilobed and male femora without modification. Similar in this respect to *S.hasta* sp. n., but differs by having: collum with 5(4)+5(4) tubercles in anterior row, 2+2 tubercles in intermediate row and 3+3 tubercles in posterior row; metaterga 2–8 with 3+3 cones in anterior row and 3+3 cones in posterior row; metaterga 9–18 with 3+3 cones in anterior row and 4+4 cones in posterior row; metatergum 19 with 3+3 tubercles/cones in anterior row and 4+4 tubercles/cones in posterior row; postfemoral part of gonopod with a triangular process and a triangular ridge.

########## Description.

SIZE. Length 26–30 mm (male), 30–32 mm (female); width of midbody metazona 2.1–2.2 mm (male), 2.7–2.9 mm (female). Width of collum = ring 2 = 3 = 4 < head = 5–17, thereafter body gradually tapering towards telson.

***Colour*** (Figure 9A, B). Specimens in life with body brown/yellowish brown; paraterga yellow; antennae (except whitish distal part of antennomeres 7 and 8), head and prozona brown/blackish brown; collum, metaterga and surface below paraterga brown/yellowish brown; sterna, epiproct and legs brown; a few basal podomeres pale brown/whitish brown.

***Antennae***. Reaching to body ring 9 or 10 (male) and 7 or 8 (female) when stretched dorsally.

***Collum*** (Figure 10A). With three transverse rows of setiferous tubercles, 5(4)+5(4) tubercles in anterior row, 2+2(1) tubercles in intermediate row and 3+3 tubercles in posterior row; with two inconspicuous setiferous notches at lateral margin; paraterga wing-like, quite short and small, tip obtuse, elevated at ca. 15°–25° (male) 10°–15° (female), directed caudolaterad.

***Tegument***. Quite dull; collum, metaterga (posterior part) and surface below paraterga coarsely microgranulate; metaterga (anterior part) smooth.

***Metaterga*** (Figure 10A, C, E). With two transverse rows of setiferous cones; metaterga 2–8 with 3+3 cones in anterior row and 3+3 cones in posterior row; metaterga 9–19 with 3(4)+3(4) cones in anterior row and 4(5)+4(5) cones in posterior row; all cones subequal in length and size.

***Paraterga*** (Figure 10A–E, H). Very long; directed almost dorsad on body rings 2–16, elevated at ca. 65°–80° (male) 60°–70° (female); directed dorsocaudad on ring 17; directed increasingly caudad on body rings 18 and 19. Ozopore visible in lateral view.

***Telson*** (Figure 10E, H, I). Epiproct quite long; tip subtruncate; lateral setiferous tubercles conspicuous; apical tubercles inconspicuous. Hypoproct subsemicircular; caudal margin round (in some specimens angular), with conspicuous setiferous tubercles.

***Sterna*** (Figs 4D; 10F, G). Sternal lobe between male coxae 4 erect, subrectangular, very long; tips emarginate, in situ directed ventrad; posterior surface bearing one pore near tip.

***Legs*** (Figs 4E, 10J). Male femora without modification.

***Gonopods*** (Figs 5B, 11). Coxa subequal in length to femur. Prefemoral part ca. 2/3 as long as femur. Femur not enlarged distally, ventrally swollen in middle part. Postfemoral part broad; mesally with a long triangular process (directed mesoanteriad) and a long triangular ridge, between process and ridge with a wide furrow. Mesal sulcus and lateral sulcus wide. Solenophore bigger and longer than postfemoral part; basally very broad: lamina lateralis long and slender, curved, tip round: lamina medialis long and slender; with two ridges in middle portion; slightly attenuated near tip; tip in situ resting very close to solenomere, terminating in small spines. Solenomere flat, curving down; tip terminating in three sharp spines, directed mesoventrad.

########## Distribution and habitat

(Figure 9C). Known only from the type locality. The specimens were found exclusively on rock walls or in caves. We have tried to find this species in other places near the type locality, but no further specimens have been collected. Given the finding only at the type locality, the new species is considered to be endemic to southern Myanmar.

########## Remarks.

No variation was found. Body ring 19 of *S.efefi* sp. n. seems to be shorter than in other species, and the tip of paraterga on collum is obtuse whereas in other species (except *S.biloba* sp. n.) it is sharp.

######### Spinaxytes hasta

Taxon classificationAnimaliaPolydesmidaParadoxosomatidae

Srisonchai, Enghoff & Panha sp. n.

http://zoobank.org/C9605402-C710-4E70-8A7B-9F41F4249C0E

**Figure 12.**
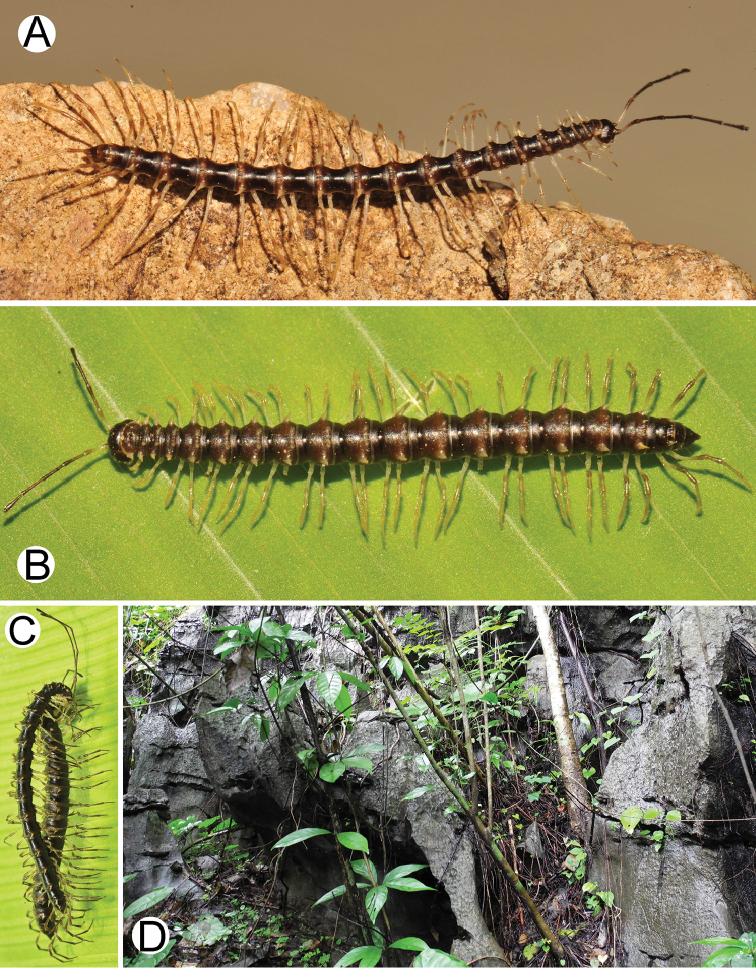
Photographs of live *Spinaxyteshasta* sp. n. and habitat **A** ♂ paratype, CUMZ-pxDGT00210 **B** ♀ paratype, CUMZ-pxDGT00210 **C** mating couple **D** habitat.

**Figure 13.**
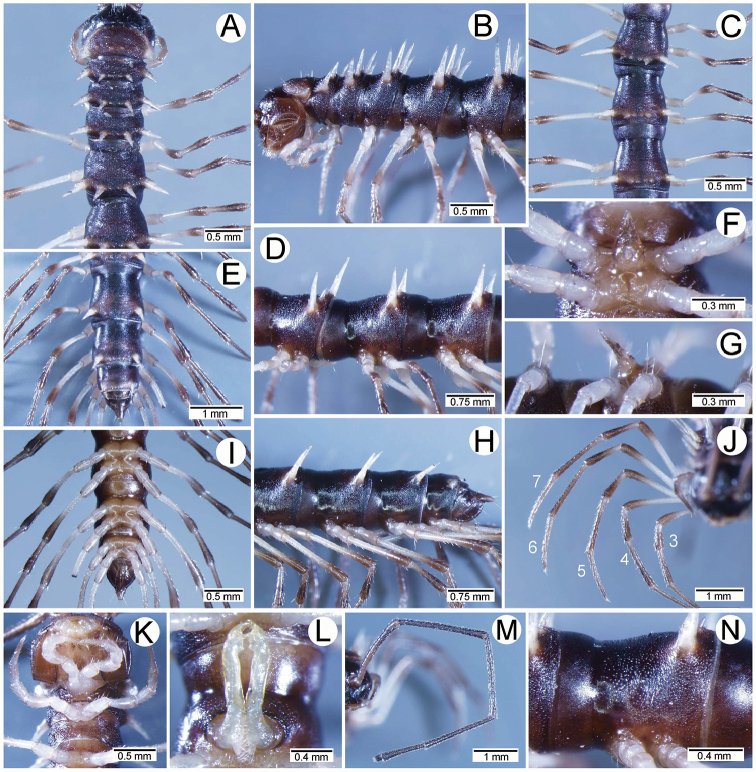
*Spinaxyteshasta* sp. n., ♂ paratype, CUMZ-pxDGT00210 **A, B** anterior body part **C, D** body rings 8–10 **E, H, I** posteriormost body rings and telson **F, G** sternal lobe between coxae 4 **J** legs 3–7 **K** legs 1–3 **L** gonopods **M** left antenna **N** sculpture of body ring 10.

**Figure 14.**
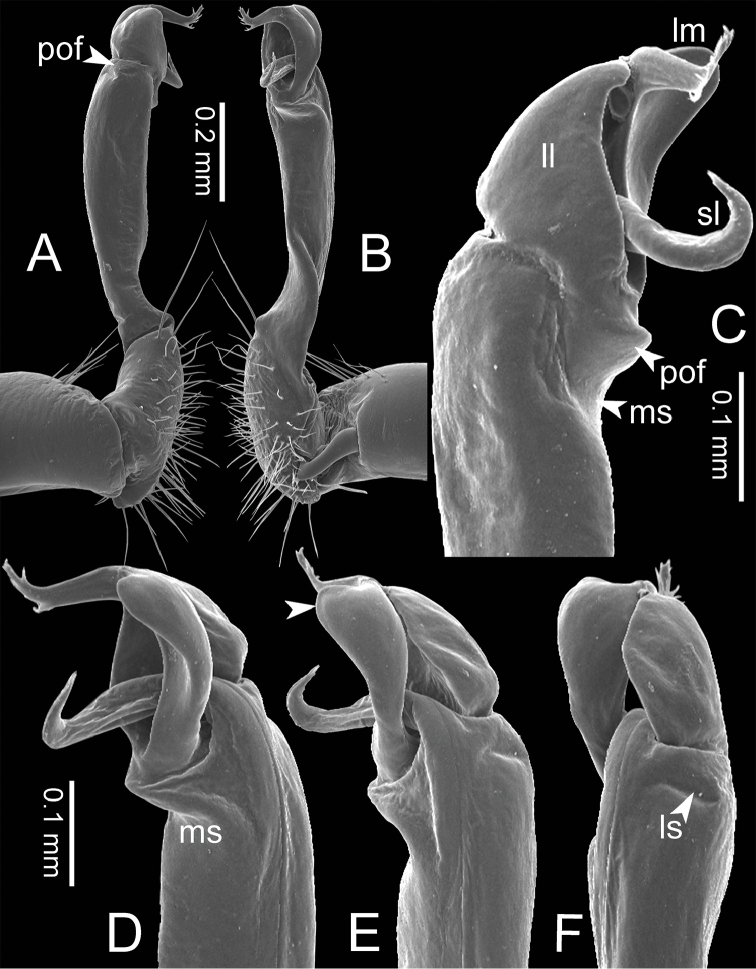
*Spinaxyteshasta* sp. n., paratype, CUMZ-pxDGT00210 – right gonopod **A** lateral view **B** mesal view **C** ventral view **D** mesodorsal view **E** dorsal view (arrowhead points to lobe on lamina medialis) **F** laterodorsal view.

Figs 4F, G ; 5C ; 12 , 13 , 14

########## Material examined.

**Holotype.** ♂, THAILAND, Chumphon Province, Thung Tako District, Khao Ma Ngaen, 10°05'27"N, 99°04'25"E, ca. 28 m a.s.l., 23 Oct. 2016, ASRU members leg. (CUMZ-pxDGT00209). **Paratypes.** 5 ♂♂, 6 ♀♀, same data as for holotype (CUMZ-pxDGT00210); 1 ♂, 1 ♀, same data as for holotype (ZMUC00040251). **Further specimens, not paratypes, all from THAILAND, Chumphon Province. Mueang Chumphon District**: 8♂♂, 1 ♀, Wat Tham Sanook, 10°28'52"N, 99°04'29"E, ca. 54 m a.s.l., 3 Jul. 2017, ASRU members leg. (CUMZ). **Lang Suan District**: 2 ♀♀, Wat Ratcha Burana School, 9°56'21"N, 99°02'26"E, ca. 34 m a.s.l., 10 Sep. 2016, ASRU members leg. (CUMZ); 1 ♂, 5 ♀♀, Wat Tham Khao Kriap (Khao Kriap Cave), 9°49'08"N, 99°02'22"E, ca. 102 m a.s.l., 5 Jun. 2009, ASRU members leg. (CUMZ). **Sawi District**: 8 ♂♂, 3 ♀♀, Wat Nam Cha, 10°17'54"N, 99°01'57"E, ca. 95 m a.s.l., 5 Jun. 2009, ASRU members leg. (CUMZ).

########## Etymology.

The name is a Latin noun in apposition meaning spear, referring to the shape of the sternal lobe between male coxae 4 which is somewhat similar to a spear.

########## Diagnosis.

Sternal lobe between male coxae 4 not bilobed, not bifurcate; male femora without modification. Similar in this respect to *S.efefi* sp. n., but differs by having: collum with 4+4 tubercles in anterior row, 1+1 tubercles in intermediate row and 2+2 tubercles in posterior row; metaterga 2–8 with 2+2 cones in anterior row and 2+2 cones in posterior row; metaterga 9–18 with 2+2 cones in anterior row and 2+2 cones in posterior row; metatergum 19 with 2+2 tubercles/cones in anterior row and 2+2 tubercles/cones in posterior row; lamina medialis (lm) with a large lobe in middle part.

########## Description.

SIZE. Length 23–33 mm (male), 26–33 mm (female); width of midbody metazona 1.7–2.2 mm (male), 2.1–2.8 mm (female). Width of collum = ring 2 = 3 = 4 < head = 5–16, thereafter body gradually tapering towards telson.

***Colour*** (Figure 12A–C). Specimens in life with body black/brownish black; paraterga white/yellowish white/whitish yellow; antennae (except whitish distal part of antennomeres 7 and 8) and metaterga (posterior part) brown/brownish black; head and collum brown/blackish brown; prozona and metaterga (anterior part) black; surface below paraterga black/brownish black; sterna and epiproct brown; legs brown/pale brown; a few basal podomeres pale whitish brown.

***Antennae*** (Figure 13M). Reaching to body ring 9 or 10 (male) and 7 (female) when stretched dorsally.

***Collum*** (Figure 13A). With three transverse rows of setiferous tubercles, 4+4 tubercles in anterior row, 1(0)+1 tubercles in intermediate row and 2+2 tubercles in posterior row; with two inconspicuous setiferous notches at lateral margin; paraterga wing-like, quite short and broad, tip sharp, elevated at ca. 10°–15° (male) 10°–15° (female), directed caudolaterad.

***Tegument***. Quite dull; collum, metaterga and surface below paraterga finely microgranulate.

***Metaterga*** (Figure 13A, C, E). With two transverse rows of setiferous tubercles/cones and spines; metaterga 2–8 with 2+2 tubercles/cones in anterior row and 2+2 spines in posterior row; metaterga 2–18 with 2+2 tubercles/cones in anterior row and 2+2 spines in posterior row; metatergum 19 with 2+2 tubercles in anterior row and 2+2 tubercles in posterior row; lateral spines of posterior row bigger and longer than mesal ones, gradually reduced in length and size on the following rings.

***Paraterga*** (Figure 13A–E, H). Very long; directed dorsolaterad on body rings 4–16, elevated at ca. 60°–70° (male) 50°–60° (female); directed caudolaterad on rings 2, 3 and 17; directed increasingly caudad on body rings 18 and 19. Ozopore visible in lateral view.

***Telson*** (Figure 13E, H, I). Epiproct long; tip subtruncate; lateral setiferous tubercles conspicuous (in some specimens inconspicuous); apical tubercles inconspicuous. Hypoproct subtrapeziform (in some specimens subsemicircular); caudal margin round (in some specimens angular), with inconspicuous setiferous tubercles.

***Sterna*** (Figs 4F; 13F, G). Sternal lobe between male coxae 4 coniform, long, spear-like; base stout; tips sharp, in situ directed almost ventrad; posterior surface bearing one pore.

***Legs*** (Figs 4G, 13J). Male femora without modification.

***Gonopods*** (Figs 5C, 14). Coxa shorter than femur. Prefemoral part ca. 2/3 as long as femur. Femur not enlarged distally, basally indented. Postfemoral part narrow. Mesal sulcus and lateral sulcus wide. Solenophore bigger and longer than postfemoral part: lamina lateralis broad and long, flattened laterally: lamina medialis long; base enlarged, slightly attenuated near the tip; middle part with a large lobe; tip a bit curving up, terminating in several small spines. Solenomere circular in transverse section, curving up, tip directed anteriad.

########## Distribution and habitat

(Figure 12D). The specimens were found on rocks or walls with plants, and some were found in a small cave. *S.hasta* sp. n. is distributed only in Chumphon Province, and we regard the new species as endemic for the Thai fauna. At Wat Nam Cha the new species coexists with *Desmoxytescervina*.

########## Remarks.

There are some variations: the lateral setiferous tubercles of the epiproct are conspicuous in some specimens, inconspicuous in others; the hypoproct is subtrapeziform in some individuals, subsemicircular in others; the caudal margin of the hypoproct is rounded in some specimens, angular in others.

######### Spinaxytes krabiensis

Taxon classificationAnimaliaPolydesmidaParadoxosomatidae

Srisonchai, Enghoff & Panha sp. n.

http://zoobank.org/DCB1E283-74DC-48F6-9876-FE1E70F8AB8A

**Figure 15.**
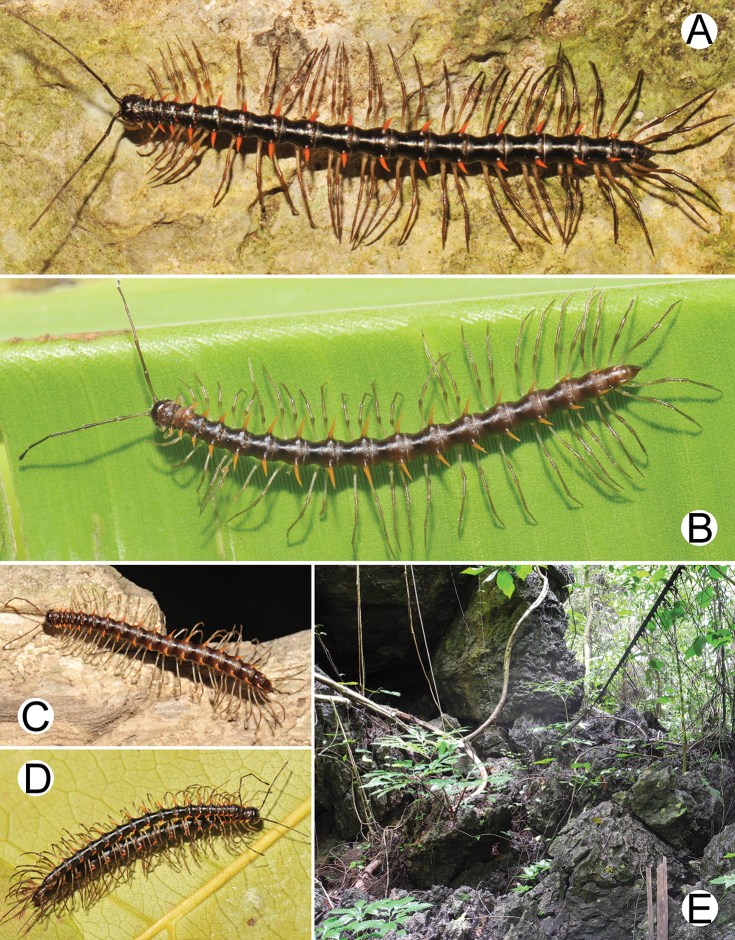
Photographs of live *Spinaxyteskrabiensis* sp. n. and habitat **A** ♂ paratype, CUMZ-pxDGT00212 **B** ♂, specimen from Tham Sa Yuan Thong (Sa Yuan Thong Cave) **C** ♀ paratype **D** mating couple **E** habitat.

**Figure 16.**
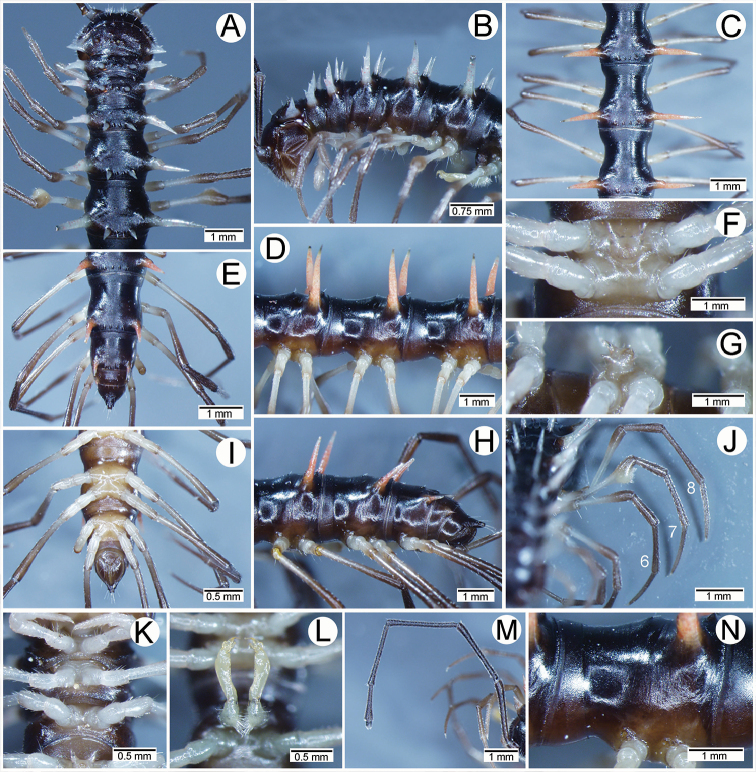
*Spinaxyteskrabiensis* sp. n., ♂ paratype, CUMZ-pxDGT00212 **A, B** anterior body part **C, D** body rings 9–11 **E, H, I** posteriormost body rings and telson **F, G** sternal lobe between coxae 4 **J** legs 4–8 **K** legs 1–3 **L** gonopods **M** right antenna **N** sculpture of body ring 10.

**Figure 17.**
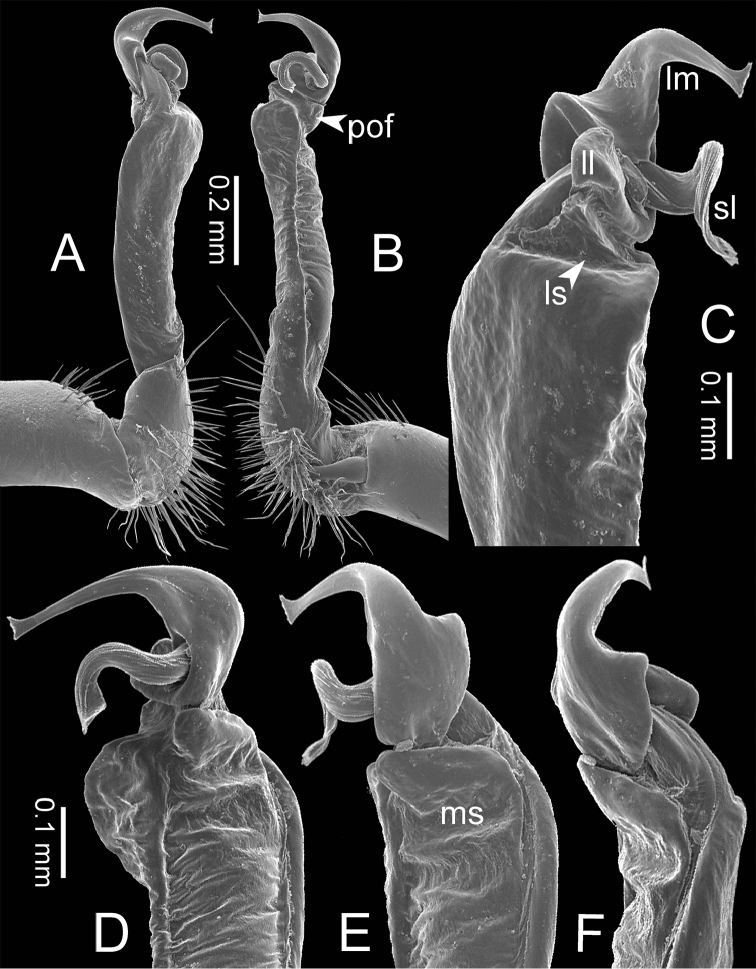
*Spinaxyteskrabiensis* sp. n., paratype, CUMZ-pxDGT00212 – right gonopod **A** lateral view **B** mesal view **C** ventral view **D** mesodorsal view **E** dorsal view **F** laterodorsal view.

Figs 3 ; 4H–J ; 5D ; 15 , 16 , 17

########## Material examined.

**Holotype.** ♂, THAILAND, Krabi Province, Mueang Krabi District, Wat Tham Sue (Tiger Cave), valley behind Tiger Cave, 8°07'38"N, 98°55'26"E, ca. 87 m a.s.l., 9 Jul. 2017, ASRU members leg. (CUMZ-pxDGT00211). **Paratypes.** 5 ♂♂, 9 ♀♀, 1 juvenile, same data as for holotype (CUMZ-pxDGT00212); 1 ♂, 1 ♀, same data as for holotype (ZMUC00040252). **Further specimens, not paratypes, all from THAILAND, Krabi Province. Ao Luek District**: 2 ♂♂, 1 ♀, Than Bok Khorani, 8°23'28"N, 98°44'07"E, ca. 46 m a.s.l., 14 Jan. 2013, ASRU members leg. (CUMZ); 5 ♂♂, 1 ♀, Than Bok Khorani, 8°23'28"N, 98°44'07"E, ca. 46 m a.s.l., 23 Aug. 2014, ASRU members leg. (CUMZ); 12 ♂♂, 7 ♀♀, 1 juvenile, Than Bok Khorani, 8°23'28"N, 98°44'07"E, ca. 46 m a.s.l., 30 Aug. 2015, ASRU members leg. (CUMZ); 3 ♂♂, Than Bok Khorani, 8°23'28"N, 98°44'07"E, ca. 46 m a.s.l., Jan. 2016, ASRU members leg. (CUMZ); 1 ♂, 5 ♀♀, P.N. Mountain Resort, 8°24'09"N, 98°44'18"E, ca. 46 m a.s.l., 30 Aug. 2015, ASRU members leg. (CUMZ); 1 ♂, 1 broken ♂, Tham Sa Yuan Thong (Sa Yuan Thong Cave), 8°23'29"N, 98°46'17"E, ca. 7 m a.s.l., 9 Oct. 2006, ASRU members leg. (CUMZ). **Muaeng Krabi District**: 1 broken ♂, Wat Tham Sue (Tiger Cave), valley behind Tiger Cave, 8°07'38"N, 98°55'26"E, ca. 87 m a.s.l., 25 Oct. 2007, ASRU members leg. (CUMZ); 1 broken ♂, 1 ♀, Wat Tham Sue (Tiger Cave), valley behind Tiger Cave, 8°07'38"N, 98°55'26"E, ca. 87 m a.s.l., 7 Oct. 2009, ASRU members leg. (CUMZ); 1 ♂, Wat Tham Sue (Tiger Cave), valley behind Tiger Cave, 8°07'38"N, 98°55'26"E, ca. 87 m a.s.l., 24 Aug. 2014, ASRU members leg. (CUMZ); 1 ♀, Wat Tham Sue (Tiger Cave), valley behind Tiger Cave, 8°07'38"N, 98°55'26"E, ca. 87 m a.s.l., 30 Aug. 2015, P. Pimvichai, P. Prasankok and N. Natarat leg. (CUMZ); 2 ♂♂, 1 ♀, 1 broken ♀, Wat Tham Sue (Tiger Cave), valley behind Tiger Cave, 8°07'38"N, 98°55'26"E, ca. 87 m a.s.l., 25 Jul. 2017, ASRU members leg. (CUMZ).

########## Etymology.

The new species is named after the province where the type locality lies.

########## Diagnosis.

Male femora 6 and 7 humped distally. Similar in this respect to *S.macaca* sp. n., but differs by having: paraterga orange, longer; male femora 6 smaller; tip of lamina lateralis round, not protuding as digitiform; tip of lamina medialis terminating in two lobes.

########## Description.

SIZE. Length 28–31 mm (male), 30–33 mm (female); width of midbody metazona 1.8–2.0 mm (male), 2.2–2.5 mm (female). Width of rings 2 = 3 = 4 < collum < head = 5–16, thereafter body gradually tapering towards telson.

***Colour*** (Figure 15A–D). Specimens in life with body black/brownish black; paraterga orange; head, antennae (except whitish distal part of antennomeres 7 and 8), collum, prozona and epiproct black; metaterga and surface below paraterga black/brownish black; sterna brown; legs brown/blackish brown; a few basal podomeres whitish brown.

***Antennae*** (Figure 16M). Reaching to body ring 8 (male) and 6 (female) when stretched dorsally.

***Collum*** (Figure 16A). With three transverse rows of setiferous tubercles/cones, 4+4 in anterior row, 1+1 in intermediate row and 2+2 in posterior row; with one conspicuous setiferous notch at lateral margin; paraterga spiniform, long, tip sharp, elevated at ca. 20°–30° in both male and female, directed caudolaterad.

***Tegument***. Quite shining; collum coarsely microgranulate; metaterga and surface below paraterga smooth.

***Metaterga*** (Figure 16A, C, E). With two transverse rows of setiferous tubercles and setiferous cones/spines; metaterga 2–7 with 2+2 tubercles in anterior row and 2+2 spines in posterior row; 8–19 with 2+2 tubercles in anterior row and 2+2 cones in posterior row; lateral cones/spines of posterior row bigger and longer than mesal ones, gradually reduced in size and length on the following rings.

***Paraterga*** (Figure 16A–E, H). Extremely long; directed dorsolaterad on body rings 2–16, elevated at ca. 45°–60° (male) 40°–50° (female), directed dorsocaudad on ring 17, directed increasingly caudad on body rings 18 and 19. Ozopore visible in subdorsal view.

***Telson*** (Figure 16E, H, I). Epiproct long; tip subtruncate; lateral setiferous tubercles mostly inconspicuous (in some specimens conspicuous); apical tubercles inconspicuous. Hypoproct subtrapeziform; caudal margin round, with inconspicuous setiferous tubercles.

***Sterna*** (Figs 4H; 16F, G). Sternal lobe between male coxae 4 bifurcate, long; tips sharp, in situ directed ventroanteriad; posterior surface bearing one pore.

***Legs*** (Figs 4I, J; 16J). Male femora 6 a bit humped; male femora 7 strongly humped.

***Gonopods*** (Figs 3, 5D, 17). Coxa subequal in length to femur. Prefemoral part ca. half as long as femur. Femur obviously enlarged distally. Postfemoral part narrow. Mesal sulcus and lateral sulcus wide. Solenophore bigger than postfemoral part: lamina lateralis small, compact, tip round: lamina medialis long; basally enlarged and slightly attenuated near the tip; tip a bit curved, terminating in two lobes. Solenomere curved and twisted, compressed in transverse section, tip directed lateroposteriad.

########## Distribution and habitat

(Figure 15E). *S.krabiensis* sp. n. inhabits Krabi Province. Considering its narrow distribution, we regard this species as endemic for the Thai fauna. It is syntopic with *Desmoxytesdelfae* (Jeekel, 1964), *Desmoxytescervina* and *Gigaxytesgigas* (Golovatch & Enghoff, 1994), which were collected from the same location (Than Bok Khorani and Wat Tham Sue (Tiger Cave)), but the new species was encountered living on rock walls or in small caves while the others were usually found on leaf litter or on tree branches.

########## Remarks.

We found variations in the lateral setiferous tubercles of the epiproct: conspicuous in some specimens, inconspicuous in others.

######### Spinaxytes macaca

Taxon classificationAnimaliaPolydesmidaParadoxosomatidae

Srisonchai, Enghoff & Panha sp. n.

http://zoobank.org/21BACA0B-8E80-43D5-8E1F-2BB1902962C2

**Figure 18.**
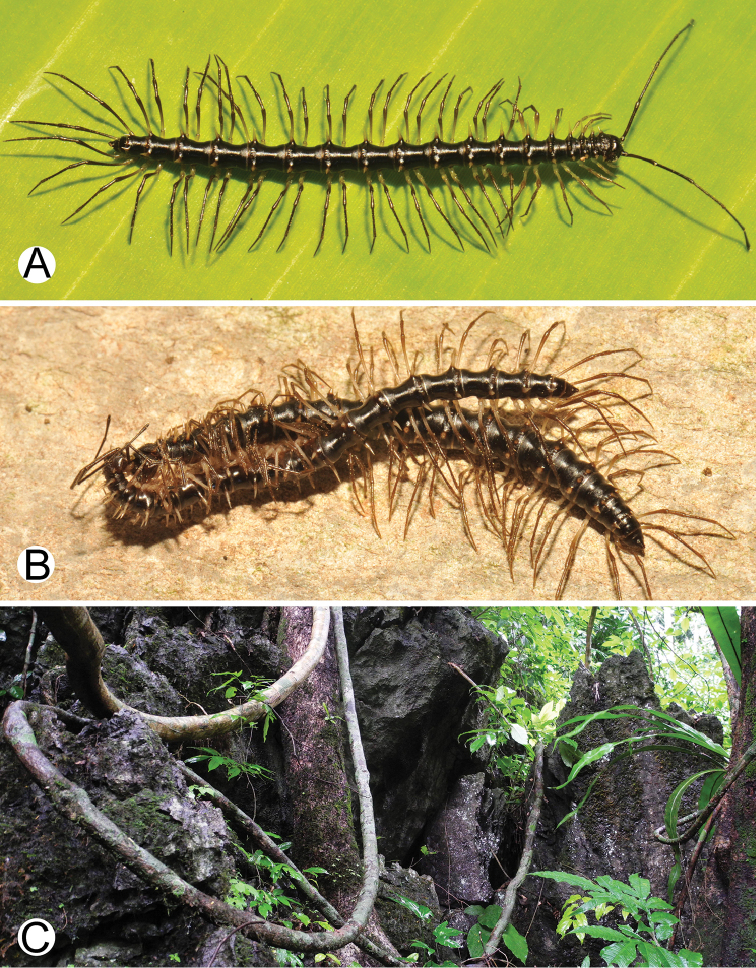
Photographs of live *Spinaxytesmacaca* sp. n. and habitat **A** ♂ paratype, CUMZ-pxDGT00214 **B** mating couple **C** habitat.

**Figure 19.**
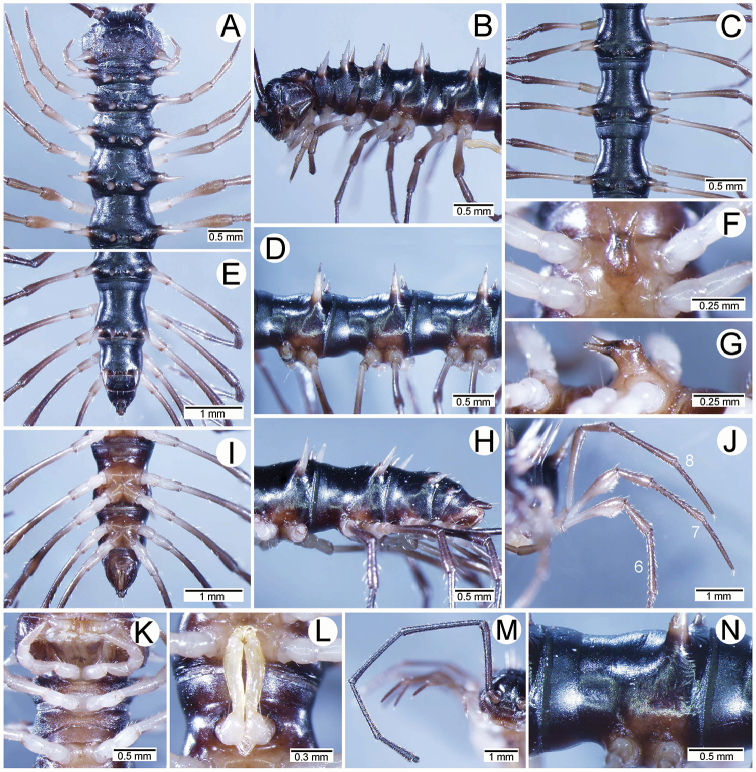
*Spinaxytesmacaca* sp. n., ♂ paratype, CUMZ-pxDGT00214 **A, B** anterior body part **C, D** body rings 8–10 **E, H, I** posteriormost body rings and telson **F, G** sternal lobe between coxae 4 **J** legs 6–8 **K** legs 1–3 **L** gonopods **M** right antenna **N** sculpture of body ring 10.

**Figure 20.**
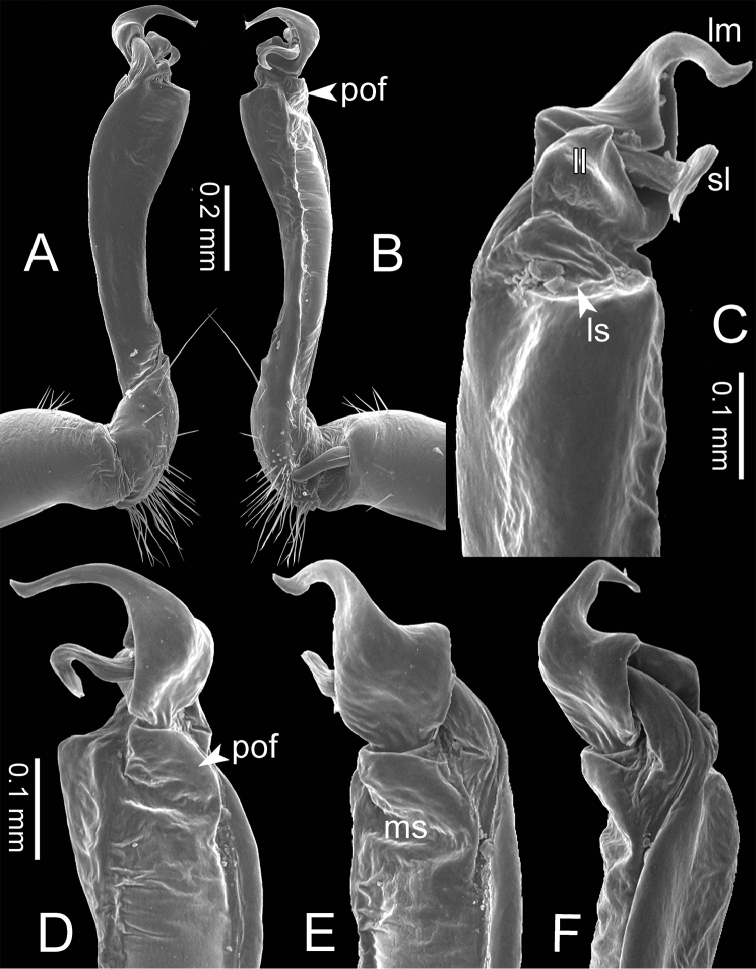
*Spinaxytesmacaca* sp. n., paratype, CUMZ-pxDGT00214 – right gonopod **A** lateral view **B** mesal view **C** ventral view **D** mesodorsal view **E** dorsal view **F** laterodorsal view.

Figs 4K–M ; 5E ; 18 , 19 , 20

########## Material examined.

**Holotype.** ♂, THAILAND, Phang Nga Province, Takua Thung District, Wat Suwan Khuha (Monkey Cave), 8°25'42"N, 98°28'22"E, ca. 27 m a.s.l., 8 Aug. 2016, ASRU members leg. (CUMZ-pxDGT00213). **Paratypes.** 7 ♂♂, 2 ♀♀, same data as for holotype (CUMZ-pxDGT00214); 1 ♂, 1 ♀, same data as for holotype (ZMUC00040253); 1 ♂ same data as for holotype (ZMUM).

########## Etymology.

The species is named after the monkey, long-tailed macaque (*Macacafascicularis*) living at the type locality (Monkey Cave).

########## Diagnosis.

Male femora 6 and 7 humped distally. Similar in this respect to *S.krabiensis* sp. n., but differs from it by having: paraterga brownish white, shorter; male femora 6 larger; tip of lamina lateralis (ll) protruding as a small lobe, digitiform; tip of lamina medialis (lm) bent, terminating in one lobe.

########## Description.

SIZE. Length 27–29 mm (male), 29–32 mm (female); width of midbody metazona 1.8–1.9 mm (male), 2.0–2.3 mm (female). Width of collum = 2 = 3 < 4 < head = 5–16, thereafter body gradually tapering towards telson.

***Colour*** (Figure 18A, B). Specimens in life with body black; paraterga brownish white; head, antennae (except whitish distal part of antennomeres 7 and 8), collum, metaterga, prozona and surface below paraterga black; sterna brown; epiproct black/brownish black; legs blackish brown; a few basal podomeres pale brown/whitish brown.

***Antennae*** (Figure 19M). Reaching to body ring 8 or 9 (male) and 7 (female) when stretched dorsally.

***Collum*** (Figure 19A). With three transverse rows of setiferous tubercles/cones, 4+4 tubercles/cones in anterior row, 1+1 tubercles/cones in intermediate row and 2+2 tubercles/cones in posterior row; with one conspicuous setiferous notch at lateral margin; paraterga spiniform, long, tip sharp, elevated at ca. 15°–20° in both male and female, directed caudolaterad.

***Tegument***. Quite shining; collum, metaterga and surface below paraterga smooth.

***Metaterga*** (Figure 19A, C, E). With two transverse rows of setiferous tubercles and cones/spines; metaterga 2–19 with 2+2 tubercles in anterior row and 2+2 cones/spines in posterior row; lateral cones/spines of posterior row bigger and longer than mesal ones, gradually reduced in length and size on the following rings.

***Paraterga*** (Figure 19A–E, H). Long; directed dorsolaterad on body rings 2–16, elevated at ca. 60°–70° (male) 50°–60° (female), directed dorsocaudad on ring 17, directed increasingly caudad on body rings 18 and 19. Ozopore visible in dorsolateral view.

***Telson*** (Figure 19E, H, I). Epiproct quite short; tip subtruncate; lateral setiferous tubercles conspicuous; apical tubercles inconspicuous. Hypoproct subtrapeziform; caudal margin round, with conspicuous setiferous tubercles.

***Sterna*** (Figs 4K; 19F, G). Sternal lobe between male coxae 4 bifurcate, long; base stout; tips very sharp, in situ directed ventroanteriad; posterior surface bearing 1 pore.

***Legs*** (Figs 4L, M; 19J). Male femora 6 and 7 humped ventrally in distal part.

***Gonopods*** (Figs 5E, 20). Coxa shorter than femur. Prefemoral part ca. half as long as femur. Femur quite enlarged distally. Postfemoral part short and narrow. Mesal sulcus and lateral sulcus wide. Solenophore longer than postfemoral part: lamina lateralis small, compact; apically protruding as a small lobe, directed mesoventrad: lamina medialis long; basally enlarged and slightly attenuated near the tip; tip bent, sharp and curving up. Solenomere curved and twisted, metazona in transverse section, tip directed posteriad.

########## Distribution and habitat

(Figure 18C). All specimens were collected in small caves near the big Monkey Cave, crawling on rock walls. It is difficult to see the new species without using a flashlight/torch as the black body colour blends in with dark rocks. This species can be found in syntopy with *Desmoxytescervina*. For the time being, *S.macaca* sp. n. is known only from the type locality and we regard it as endemic to Thailand.

########## Remarks.

*S.macaca* sp. n. is morphologically similar to *S.krabiensis* sp. n. and *S.uncus* sp. n. with which it shares a fork-like sternal lobe between male coxae 4 and a small lamina lateralis.

######### Spinaxytes palmata

Taxon classificationAnimaliaPolydesmidaParadoxosomatidae

Srisonchai, Enghoff & Panha sp. n.

http://zoobank.org/2A4B8447-3443-44D7-B463-0A6A2AB840C9

**Figure 21.**
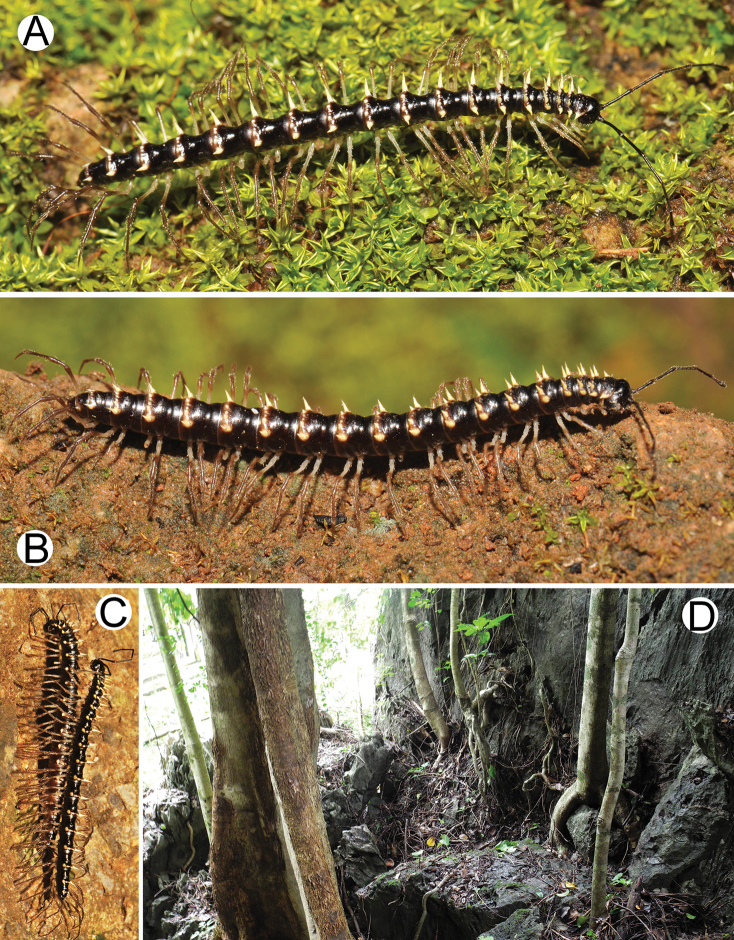
Photographs of live *Spinaxytespalmata* sp. n. and habitat **A** ♂ paratype, CUMZ-pxDGT00216 **B** ♀ paratype, CUMZ-pxDGT00216 **C** mating couple **D** habitat.

**Figure 22.**
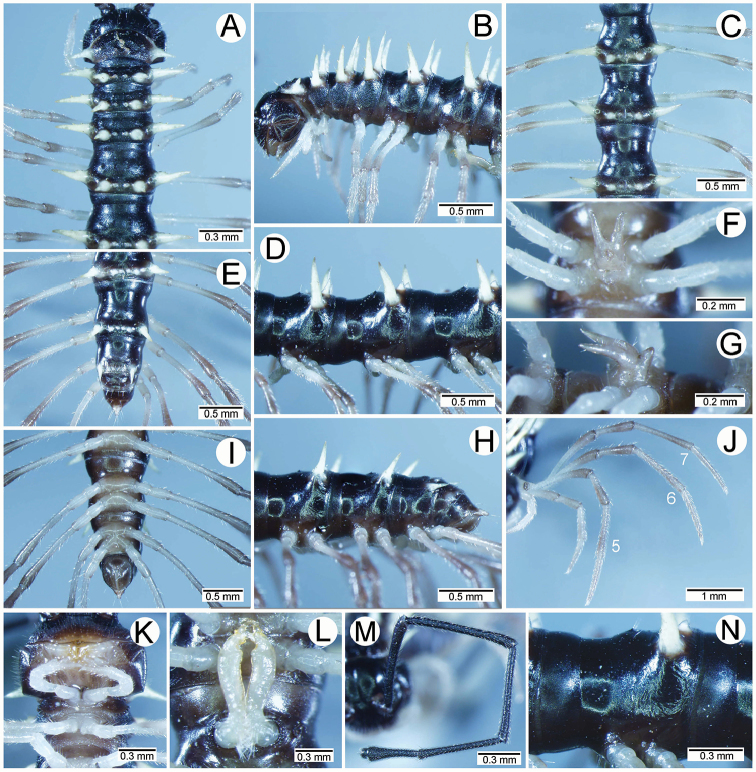
*Spinaxytespalmata* sp. n., ♂ paratype, CUMZ-pxDGT00216 **A, B** anterior body part **C, D** body rings 8–10 **E, H, I** posteriormost body rings and telson **F, G** sternal lobe between coxae 4 **J** legs 5–7 **K** legs 1–3 **L** gonopods **M** left antenna **N** sculpture of body ring 10.

**Figure 23.**
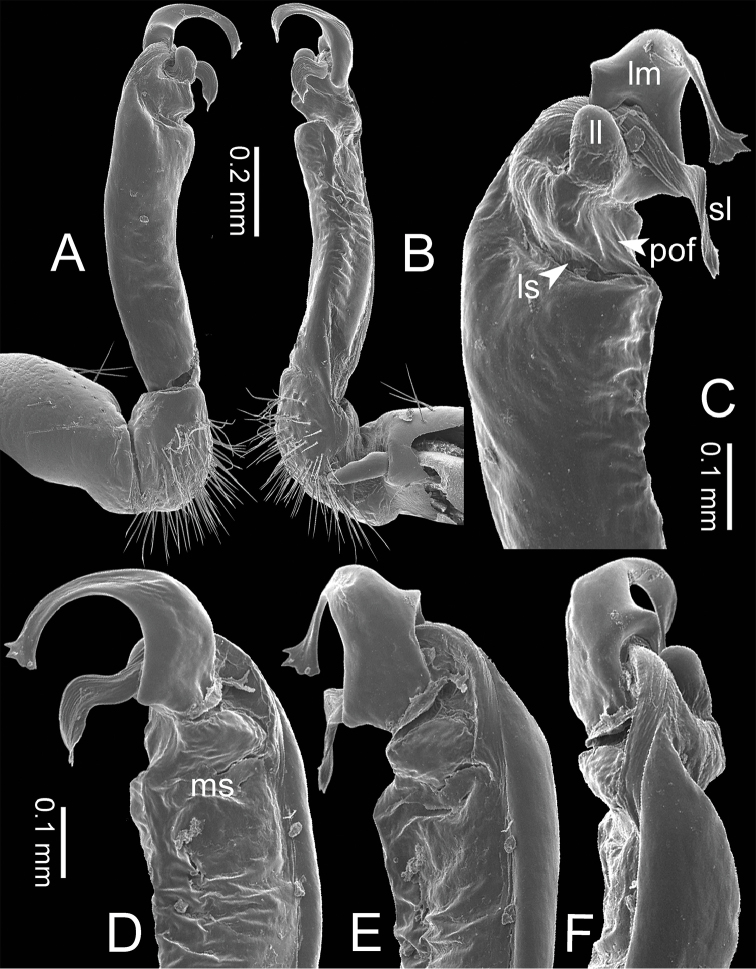
*Spinaxytespalmata* sp. n., paratype, CUMZ-pxDGT00216 – right gonopod **A** lateral view **B** mesal view **C** ventral view **D** mesodorsal view **E** dorsal view **F** laterodorsal view.

Figs 2 ; 4N, O ; 5F ; 21 , 22 , 23

########## Material examined.

**Holotype.** ♂, THAILAND, Surat Thani Province, Phanom District, Wat Tham Wararam, 8°53'07"N, 98°40'01"E, ca. 51 m a.s.l., 6 Aug. 2016, ASRU members leg. (CUMZ-pxDGT00215). **Paratypes.** 23 ♂♂, 22 ♀♀, same data as for holotype (CUMZ-pxDGT00216); 1 ♂, 1 ♀, same data as for holotype (ZMUC00040254); 1 ♂, 1 ♀, same data as for holotype (ZMUM); 1 ♂, 1 ♀, same data as for holotype (NHMW9425); 1 ♂, 1 ♀, same data as for holotype (NHMUK). **Further specimens, not paratypes.** THAILAND: 1 ♂, 2 ♀♀, Surat Thani Province, Phanom District, Wat Tham Wararam, 8°53'07"N, 98°40'01"E, ca. 51 m a.s.l., 5 Aug. 2014, ASRU members leg. (CUMZ).

########## Etymology.

The species name is a Latin adjective, referring to the tip of lamina medialis which is somewhat hand-shaped.

########## Diagnosis.

Male femora without modification. Similar in this respect to *S.efefi* sp. n., *S.hasta* sp. n., *S.sutchariti* sp. n. and *S.tortioverpa* sp. n., but differs from them by having: anterior part of sternal lobe between male coxae 4 bifurcate, fork-like; tip of lamina medialis expanded, hand-shaped.

########## Description.

SIZE. Length 26–30 mm (male), 27–32 mm (female); width of midbody metazona 1.9–2.2 mm (male), 2.0–2.4 mm (female). Width of collum = 2 = 3 = 4 < head < 5–16, thereafter body gradually tapering towards telson.

***Colour*** (Figure 21A–C). Specimens in life with body black; paraterga orange; head, antennae (except whitish distal part of antennomeres 7 and 8), collum, prozona, metaterga (except white spines in posterior row) and surface below paraterga black; sterna and legs brown; epiproct pale brown; a few basal podomeres whitish brown.

***Antennae*** (Figure 22M). Reaching to body ring 8 (male) and 6 or 7 (female) when stretched dorsally.

***Collum*** (Figure 22A). With three transverse rows of setiferous tubercles/cones, 4+4 in anterior row, 1(0)+1(0) in intermediate row and 2(1)+2(1) in posterior row; with one conspicuous setiferous notch at lateral margin; paraterga spiniform, long, tip sharp, elevated at ca. 15°–20° (male) 10°–15° (female), directed almost laterad.

***Tegument***. Very shining; collum coarsely microgranulate; metaterga and surface below paraterga smooth.

***Metaterga*** (Figure 22A, C, E). With two transverse rows of setiferous cones and setiferous spines; metaterga 2–19 with 2+2 cones in anterior row and 2+2 spines in posterior row; lateral cones/spines of posterior row bigger and longer than mesal ones, gradually reduced in size and length on the following rings.

***Paraterga*** (Figure 22A–E, H). Very long; directed dorsolaterad on body rings 2–17, elevated at ca. 50°–60° (male) 45°–60° (female), directed increasingly caudad on body rings 18 and 19. Ozopore visible in dorsolateral view.

***Telson*** (Figure 22E, H, I). Epiproct quite short; tip subtruncate; lateral setiferous tubercles conspicuous; apical tubercles inconspicuous. Hypoproct subtrapeziform (in some specimens subtriangular); caudal margin round (in some specimens angular), with inconspicuous setiferous tubercles.

***Sterna*** (Figs 4N; 22F, G). Sternal lobe between male coxae 4 with two parts; anterior part bifurcate, tuning-fork-like, long, tips sharp, in situ directed ventroanteriad; posterior margin of anterior part bearing 1 pore; posterior part swollen, short.

***Legs*** (Figs 4O, 22J). Male femora without modification.

***Gonopods*** (Figs 5F, 23). Coxa shorter than femur. Prefemoral part ca. half as long as femur. Femur not enlarged distally. Postfemoral part broad. Mesal sulcus and lateral sulcus wide. Solenophore a bit bigger than postfemoral part: lamina lateralis small, oval, tip round: lamina medialis long; basally enlarged and slightly attenuated near the tip; tip fringed, hand-shaped; tip curving down, in situ resting close to solenomere. Solenomere curved and twisted, compressed in transverse section, tip directed posteriad.

########## Distribution and habitat

(Figure 21D). *S.palmata* sp. n. is known only from the type locality. We regard this species as endemic for the Thai fauna. The new species can be found in syntopy with *Desmoxytescorythosaurus* Srisonchai, Enghoff & Panha, 2018, crawling on humid rock walls.

########## Remarks.

There are variations in the hypoproct: subtrapeziform in some specimens, subtriangular in the others; caudal margin in some individuals round, angular in the others.

######### Spinaxytes sutchariti

Taxon classificationAnimaliaPolydesmidaParadoxosomatidae

Srisonchai, Enghoff & Panha sp. n.

http://zoobank.org/4E54ED20-66AB-4BF7-B40D-D6F6699CF282

**Figure 24.**
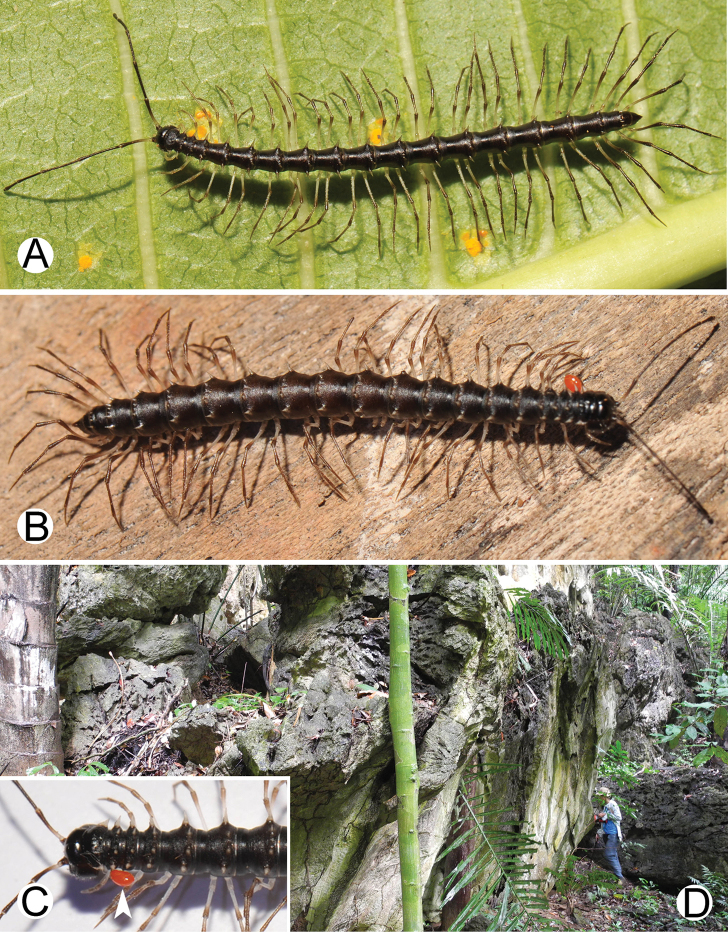
Photographs of live *Spinaxytessutchariti* sp. n. and habitat **A** ♂ paratype, CUMZ-pxDGT00218 **B** ♀ paratype, CUMZ-pxDGT00218 **C** parasitic mite (arrowhead) **D** habitat.

**Figure 25.**
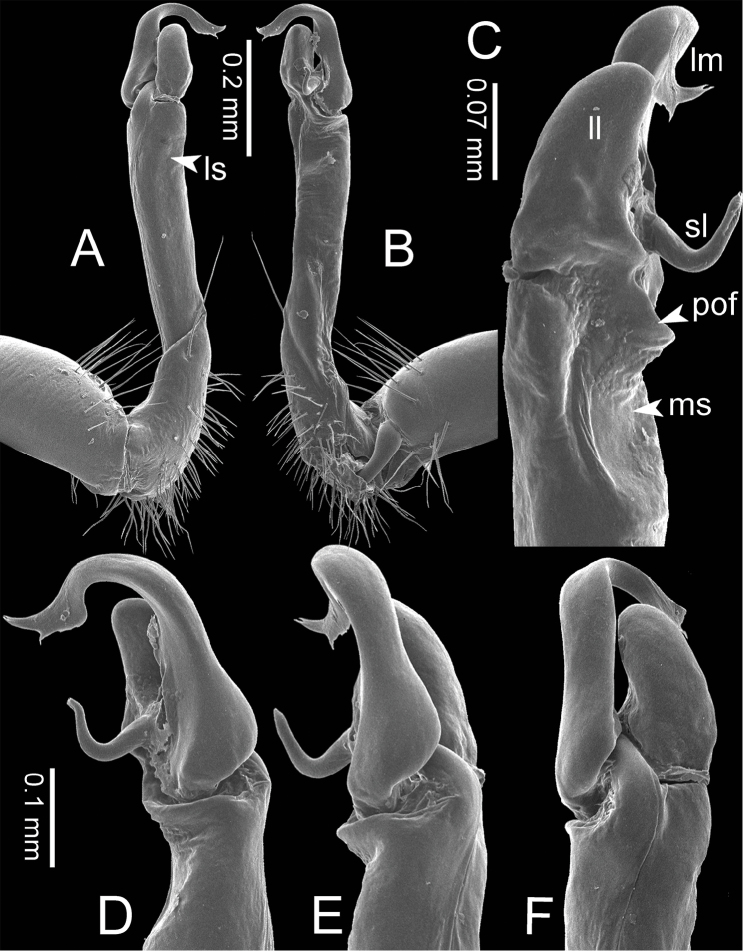
*Spinaxytessutchariti* sp. n., paratype, CUMZ-pxDGT00218 – right gonopod **A** lateral view **B** mesal view **C** ventral view **D** mesodorsal view **E** dorsal view **F** laterodorsal view.

Figs 4P, Q ; 5G ; 24 ; 25

########## Material examined.

**Holotype.** ♂, THAILAND, Krabi Province, Muang Krabi District, Tham Na Mee (Na Mee Cave), 8°08'12"N, 98°48'23"E, ca. 70 m a.s.l., 31 Aug. 2015, C. Sutcharit leg. (CUMZ-pxDGT00217). **Paratypes.** 7 ♂♂, 4 ♀♀, same data as for holotype (CUMZ-pxDGT00218).

########## Etymology.

The name honours associate professor Dr. Chirasak Sutcharit, malacologist of ASRU (CUMZ), collector of this new species and numerous other dragon millipedes.

########## Diagnosis.

Male femora without modification, sternal lobe between male coxae 4 incompletely bilobed. Similar in these respects to *S.palmata* sp. n., but differs by having: a large and round lamina lateralis; tip of lamina medialis terminating in two spines; distal part of solenomere circular in tranverse section.

########## Description.

SIZE. Length 20–25 mm (male), 23–27 mm (female); width of midbody metazona 1.5–1.8 mm (male), 1.9–2.2 mm (female). Width of collum = 2 = 3 = 4 < head = 5–16, thereafter body gradually tapering towards telson.

***Colour*** (Figure 24A–C). Specimens in life with body black; paraterga brownish white; head, antennae (except whitish distal part of antennomeres 7 and 8), collum, prozona, metaterga and epiproct black; surface below paraterga black/brownish black; sterna brown; legs brown/blackish brown; a few basal podomeres whitish brown.

***Antennae***. Reaching to body ring 8 or 9 (male) and 6 (female) when stretched dorsally.

***Collum***. With three transverse rows of setiferous tubercles, 4+4 tubercles in anterior row, 1+1 tubercles in intermediate row and 2+2 tubercles in posterior row; with one inconspicuous setiferous notch at lateral margin; paraterga spiniform, quite short, tip sharp, elevated at ca. 15°–20° in both male and female, directed caudolaterad.

***Tegument***. Quite dull; collum and metaterga (posterior part) coarsely microgranulate; metaterga (anterior part) and surface below paraterga smooth.

***Metaterga***. With two transverse rows of setiferous tubercles and setiferous spines; metaterga 2–19 with 2+2 tubercles in anterior row and 2+2 spines in posterior row; lateral spines of posterior row bigger and longer than mesal ones, subequal in size and length on all body rings.

***Paraterga***. Long; directed dorsolaterad on body rings 2–16, elevated at ca. 45°–50° (male) 40°–50° (female), directed dorsocaudad on ring 17, directed increasingly caudad on body rings 18 and 19. Ozopore visible in lateral view.

***Telson***. Epiproct quite long; tip subtruncate; lateral setiferous tubercles conspicuous; apical tubercles inconspicuous. Hypoproct subtrapeziform (in some specimens subtriangular); caudal margin round (in some specimens angular), with inconspicuous setiferous tubercles.

***Sterna*** (Figure 4P). Sternal lobe between male coxae 4 incompletely bilobed; tips sharp, in situ directed laterad; posterior surface bearing 2 pores.

***Legs*** (Figure 4Q). Male femora without modification.

***Gonopods*** (Figs 5G, 25). Coxa subequal in length to femur. Prefemoral part ca. 2/3 as long as femur. Femur not enlarged distally. Postfemoral part narrow. Mesal sulcus and lateral sulcus wide. Solenophore bigger and longer than postfemoral part: lamina lateralis oval, large, long, tip round: lamina medialis long and slender; base enlarged, slightly attenuated near the tip; tip curving down, with two sharp spines (one smaller, one bigger). Solenomere curving up, circular in transverse section, tip directed anteriad.

########## Distribution and habitat

(Figure 24D). *S.sutchariti* sp. n. is known only from the type locality, and we regard it as endemic to Thailand. The new species can be found in the same area as *Gigaxytesgigas*, but we assume that they live in different microhabitats: *G.gigas* was collected from the ground in leaf litter, whereas the new species was found on humid rock walls.

########## Remarks.

We found variation in the hypoproct: in some specimens subtrapeziform, in others subtriangular; caudal margin in some individuals round, in others angular. Parasitic mite larvae, probably of the genus *Leptus* Latreille, 1796, were found attached to the anterior body part of some female specimens. Larvae of ?*Leptus* have previously been found on species of *Desmoxytes* (*D.cervina*) and *Nagaxytes* (*N.acantherpestes* (Golovatch & Enghoff, 1994)) (Srisonchai et al. 2018a, 2018b, see also Southcott 1992).

######### Spinaxytes tortioverpa

Taxon classificationAnimaliaPolydesmidaParadoxosomatidae

Srisonchai, Enghoff & Panha sp. n.

http://zoobank.org/97E5F638-2A4D-4834-B93C-838548BAD087

**Figure 26.**
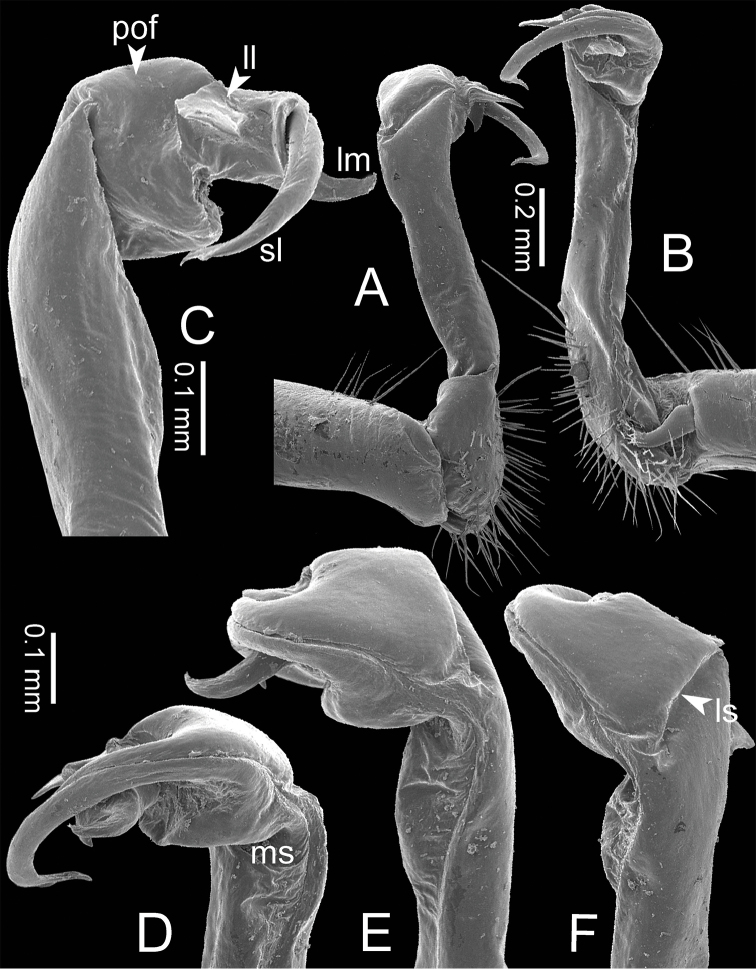
*Spinaxytestortioverpa* sp. n., paratype, CUMZ-pxDGT00220 – right gonopod **A** lateral view **B** mesal view **C** ventral view **D** mesodorsal view **E** dorsal view **F** laterodorsal view.

Figs 1 ; 4R, S ; 5H ; 26

########## Material examined.

**Holotype.** ♂, MALAYSIA, Perak State, Ipoh City, Gua Tempurung, limestone mountain, 4°24'58"N, 101°11'16"E, ca. 92 m a.s.l., 27 Sep. 2007, B. W. Ng and ASRU members leg. (CUMZ-pxDGT00219). **Paratypes.** 1 ♂, 1 ♂ gonopods missing, 1 ♀, same data as for holotype (CUMZ-pxDGT00220).

########## Etymology.

The name is a noun in apposition, from the Latin *tortio* meaning torsion and *verpa* for penis, refers to the distal part of gonopod (postfemoral part, solenophore and solenomere) which makes a 90 degrees torsion relative to the femoral part.

########## Diagnosis.

Male femora without modification; collum with 4+4 tubercles in anterior row, 1+1 tubercles in intermediate row and 2+2 tubercles in posterior row. Similar in these respects to *S.hasta* sp. n. and *S.palmata* sp. n., but differs from them by having: a completely bilobed sternal lobe between male coxae 4; postfemoral part, solenophore and solenomere angled 90 degrees with femoral part; lamina lateralis with two lobes – the lateral one spine-like, the mesal one shorter and ridge-like; solenomere very long, longer than lamina medialis.

########## Description.

SIZE. Length 28–30 mm (male), 30–33 mm (female); width of midbody metazona 2.0 mm (male), 2.8 mm (female). Width of rings 2 = 3 = 4 < head = 5–16, thereafter body gradually tapering towards telson.

***Colour***. Specimens in life with body black/brownish black. Colour in alcohol: after 10 years changed to brown; paraterga brownish white; antennae brown (except whitish distal part of antennomeres 7 and 8); head, collum, metaterga and prozona blackish brown; surface below paraterga brown/blackish brown; sterna, epiproct and legs pale brown; a few basal podomeres whitish brown.

***Antennae***. Reaching to body ring 8 or 9 (male) and 6 or 7 (female) when stretched dorsally.

***Collum***. With three transverse rows of setiferous tubercles, 4+4 tubercles in anterior row, 1+1 tubercles in intermediate row and 2+2 tubercles in posterior row; anterior margin truncate; with one inconspicuous setiferous notch at lateral margin; paraterga wing-like, long and broad, tip sharp, elevated at ca. 15°–20° in both male and female, directed caudolaterad.

***Tegument***. Quite dull; collum, metaterga and surface below paraterga finely microgranulate.

***Metaterga***. With two transverse rows of setiferous tubercles/cones and spines; metaterga 2–8 with 2+2 cones in anterior row and 2+2 spines in posterior row; metaterga 9–19 with 2+2 tubercles/cones in anterior row and 2+2 spines in posterior row; mesal spines of posterior row bigger and longer than lateral ones, gradually reduced in length and size on posterior rings.

***Paraterga***. Very long; directed dorsolaterad on body rings 2–17, elevated at ca. 65°–70° (male) 60°–70° (female), directed increasingly caudad on body rings 18 and 19. Ozopore visible in dorsolateral view.

***Telson***. Epiproct quite short; tip subtruncate; lateral setiferous tubercles inconspicuous; apical tubercles inconspicuous. Hypoproct subsemicircular; caudal margin round, with inconspicuous setiferous tubercles.

***Sterna*** (Figure 4R). Sternal lobe between male coxae 4 completely divided into two lobes, long, spine-like; tips in situ directed ventrad; posterior surface a bit swollen, bearing 2 pores.

***Legs*** (Figure 4S). Male femora without modification.

***Gonopods*** (Figs 5H, 26). Coxa subequal in length to femur. Prefemoral part about 2/3 as long as femur. Femur quite enlarged distally. Postfemoral part large, broad and wide; angled 90 degrees with femur. Mesal sulcus and lateral sulcus wide. Solenophore smaller than postfemoral part: lamina lateralis apparently with two lobes demarcated from each other; lateral lobe very long, process-like, its tip in situ directed ventrad; mesal lobe short and wide, supporting solenomere: lamina medialis long, base not enlarged, tip directed mesad. Solenomere obviously longer than lamina medialis, circular in transverse section, curving down, tip directed laterad.

########## Distribution and habitat.

Known only from the type locality which is currently a tourist attraction (cave). We regard this species as endemic to Malaysia.

########## Remarks.

A photograph of a live specimen was not taken during the field survey, but our collector noticed its black or brownish black colour. All specimens were seen crawling on rock walls where they seem to blend perfectly with the substrate. No variation in morphological characters was found.

######### Spinaxytes uncus

Taxon classificationAnimaliaPolydesmidaParadoxosomatidae

Srisonchai, Enghoff & Panha sp. n.

http://zoobank.org/1C0878B4-7450-472D-A495-4C75B44CA4E0

**Figure 27.**
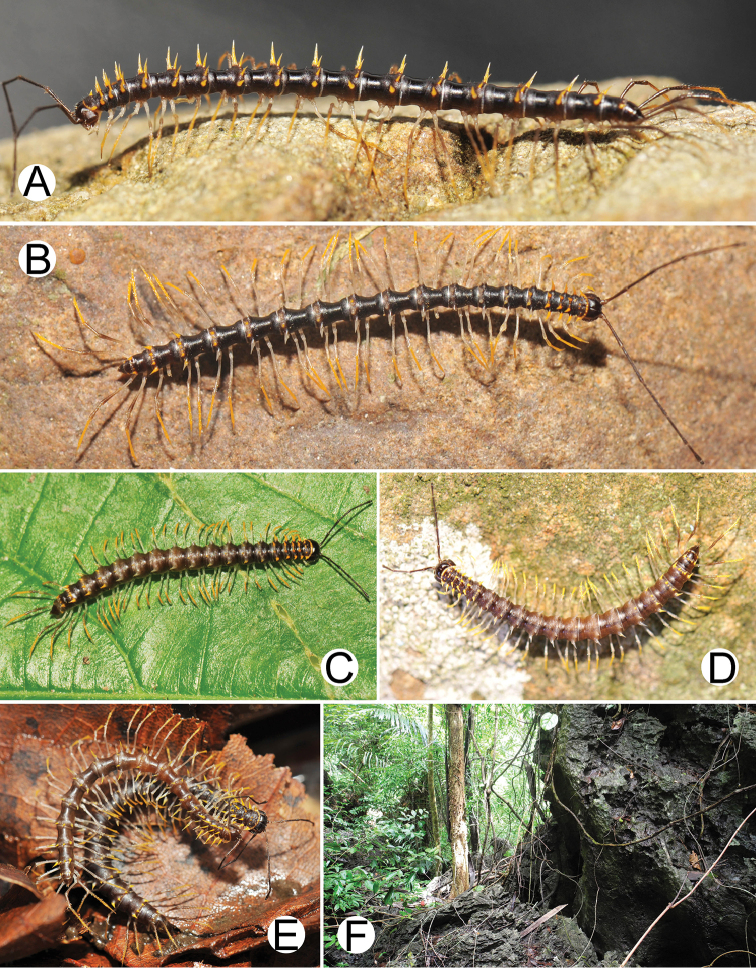
Photographs of live *Spinaxytesuncus* sp. n. and habitat **A** ♂ paratype, CUMZ-pxDGT00222 **B** ♂, specimen from Wat Kerewong (Tham Koab) **C** ♀ paratype, CUMZ-pxDGT00222 **D** ♀, specimen from Wat Kerewong (Tham Koab) **E** mating couple **F** habitat.

**Figure 28.**
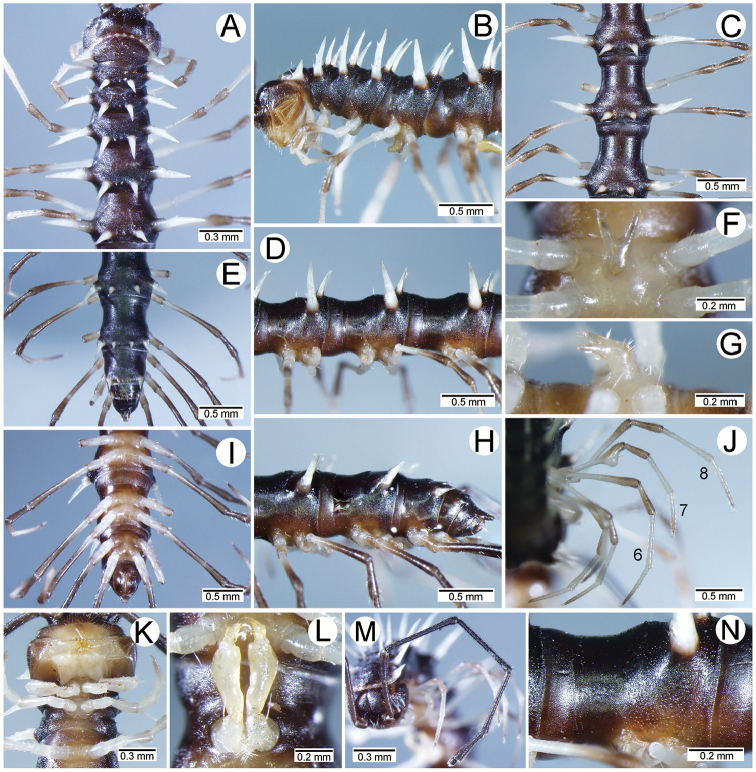
*Spinaxytesuncus* sp. n., ♂ paratype, CUMZ-pxDGT00222 **A, B** anterior body part **C, D** body rings 8–10 **E, H, I** posteriormost body rings and telson **F, G** sternal lobe between coxae 4 **J** legs 6–8 **K** legs 1–3 **L** gonopods **M** left antenna **N** sculpture of body ring 10.

**Figure 29.**
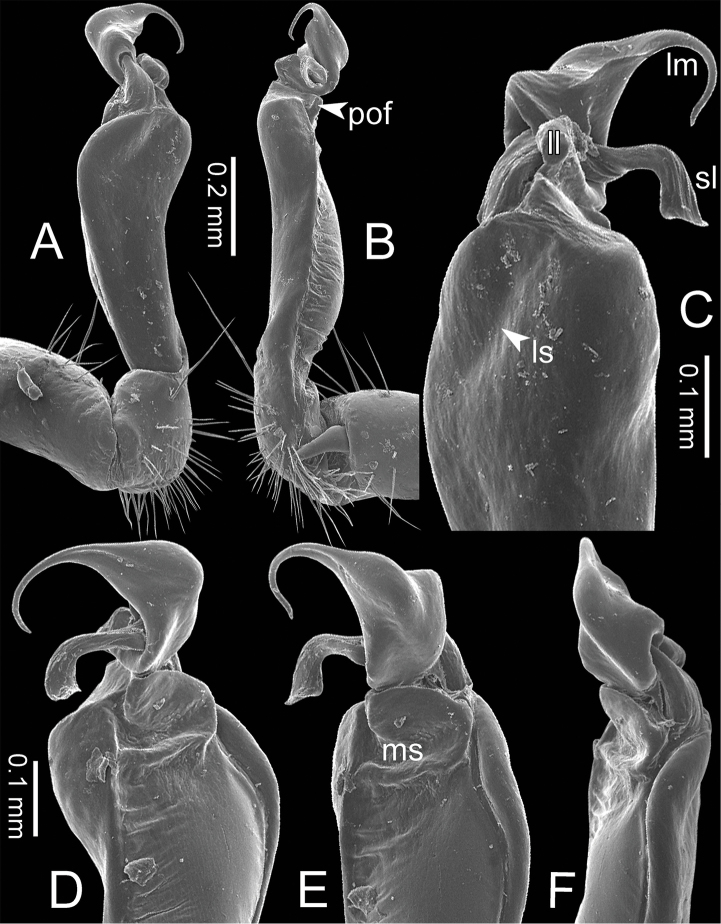
*Spinaxytesuncus* sp. n., paratype, CUMZ-pxDGT00222 – right gonopod **A** lateral view **B** mesal view **C** ventral view **D** mesodorsal view **E** dorsal view **F** laterodorsal view.

Figs 4T–V ; 5I ; 27 , 28 , 29

########## Material examined.

**Holotype.** ♂, THAILAND, Phang Nga Province, Mueang Phang Nga District, Phung Chang Cave, 8°26'34"N, 98°30'59"E, ca. 24 m a.s.l., 8 Aug. 2016, ASRU members leg. (CUMZ-pxDGT00221). **Paratypes.** 15 ♂♂, 16 ♀♀, same data as for holotype (CUMZ-pxDGT00222); 1 ♂, 1 ♀, same data as for holotype (ZMUC00040255); 1 ♂, 1 ♀, same data as for holotype (ZMUM); 1 ♂, 1 ♀, same data as for holotype (NHMW9424). **Further specimens, not paratypes, all from THAILAND, Phang Nga Province. Muaeng Phang Nga District**: 4 ♂♂, 2 ♀♀, Phung Chang Cave, 8°26'34"N, 98°30'59"E, ca. 24 m a.s.l., 6 Aug. 2014, ASRU members leg. (CUMZ); 4 ♂♂, Phung Chang Cave, 8°26'34"N, 98°30'59"E, ca. 24 m a.s.l., 5 Aug. 2015, ASRU members leg. (CUMZ); 2 ♂♂, 4 broken ♀♀, Tham Nam Pud, 8°27'50"N, 98°32'36"E, ca. 58 m a.s.l., 8 Oct. 2006, ASRU members leg. (CUMZ); 3 ♂♂, 2 ♀♀, Tham Nam Pud, 8°27'50"N, 98°32'36"E, ca. 58 m a.s.l., 5 Aug. 2015, ASRU members leg. (CUMZ); 2 ♂♂, 1 ♀, Wat Tham Bang Toei, 8°27'52"N, 98°34'10"E, ca. 24 m a.s.l., 10 Jul. 2017, ASRU members leg. (CUMZ); 8 ♂♂, 2 ♀♀, Tham Pha Phueng Bureau of Monks, 8°28'24"N, 98°32'15"E, ca. 78 m a.s.l., 10 Jul. 2017, ASRU members leg. (CUMZ). **Thap Put District**: 11 ♂♂, 3 ♀♀, Wat Kerewong (Tham Koab), 8°31'52"N, 98°34'39"E, ca. 76 m a.s.l., 9 Jul. 2017, ASRU members leg. (CUMZ).

########## Etymology.

The name is a Latin noun in apposition (*uncus*), meaning hook, and refers to the hook-like lamina medialis of gonopod.

########## Diagnosis.

Differs from other species by having only male femora 7 strongly humped distally, in combination with the distal part of lamina medialis hook-like, tip long and sharp.

########## Description.

SIZE. Length 20–27 mm (male), 25–29 mm (female); width of midbody metazona 1.4–1.6 mm (male), 2.1–2.3 mm (female). Width of collum < 2 = 3 < 4 < head = 5–16, thereafter body gradually tapering towards telson.

***Colour*** (Figure 27A–E). Specimens in life with body black; paraterga yellow/whitish yellow; head, antennae (except whitish distal part of antennomeres 7 and 8) and prozona black; collum, metaterga and surface below paraterga black/brownish black; sterna and epiproct brown; legs yellow; a few basal podomeres white.

***Antennae*** (Figure 28M). Reaching to body ring 8 or 9 (male) and 6 or 7 (female) when stretched dorsally.

***Collum*** (Figure 28A). With three transverse rows of setiferous cones, 4+4 in anterior row, 1+1 in intermediate row and 2+2 in posterior row; with two conspicuous setiferous notches at lateral margin (first notch located at base of paraterga very close to cones of anterior row; paraterga spiniform, long, tip sharp, elevated at ca. 20°–30° in both male and female, directed almost laterad.

***Tegument***. Quite shining; collum, metaterga (posterior part) and surface below paraterga finely microgranulate; metaterga (anterior part) coarsely microgranulate.

***Metaterga*** (Figure 28A, C, E). With two transverse rows of setiferous cones and setiferous spines; metaterga 2–19 with 2+2 cones in anterior row and 2+2 spines in posterior row; lateral spines of posterior row very long, bigger and longer than mesal ones.

***Paraterga*** (Figure 28A–E, H). Very long; directed dorsolaterad on body rings 2–16, elevated at ca. 40°–50° (male) 40°–45° (female), directed dorsocaudad on ring 17, directed increasingly caudad on body rings 18 and 19. Ozopore visible in dorsolateral view.

***Telson*** (Figure 28E, I, H). Epiproct short; tip subtruncate; lateral setiferous tubercles inconspicuous; apical tubercles inconspicuous. Hypoproct subtrapeziform (in some specimens subsemicircular); caudal margin round, with conspicuous setiferous tubercles (in some specimens inconspicuous).

***Sterna*** (Figs 4T; 28F, G). Sternal lobe between male coxae 4 bifurcate, long; tips sharp, in situ directed ventroanteriad; posterior surface bearing one pore.

***Legs*** (Figs 5U, V; 28J). Male femora 7 strongly humped ventrally in distal part.

***Gonopods*** (Figs 5I, 29). Coxa subequal in length to femur. Prefemoral part almost half as long as femur. Femur obviously enlarged distally. Postfemoral part small, narrow. Mesal sulcus wide; lateral sulcus narrow. Solenophore bigger and longer than postfemoral part: lamina lateralis very small, compact, tip round: lamina medialis long; basally enlarged and slightly attenuated near the tip; apically sharp, long, hook-like; tip curving down, in situ resting close to solenomere. Solenomere curved and twisted, compressed in transverse section, tip directed posteriad.

########## Distribution and habitat

(Figure 27F). Known only from Phang Nga Province; we regard *S.uncus* sp. n. as endemic for the Thai fauna. Most specimens were found on rock walls near the cave, some were seen crawling on leaf litter on the rock. The new species has been encountered in syntopy with *Desmoxytescervina* at Phung Chang Cave, Tham Nam Pud and Wat Kerewong (Tham Koab).

########## Remarks.

Some variation in the hypoproct was observed in this species: in some specimens subtrapeziform, in others subsemicircular; caudal margin in some individuals conspicuous, in others inconspicuous. In addition, specimens from Wat Kerewong (Tham Koab) have smaller and shorter paraterga than other specimens.

## Discussion

The new genus *Spinaxytes*, defined by Srisonchai et al. (2018a) as the “spiny” group of dragon millipedes, at that time without described members, adds to the challenge of understanding the patterns of paratergal and gonopod evolution in the dragon millipedes. The nine species, all new, described here are recorded from Malaysia, Myanmar, and Thailand. They are united in the new genus by sharing the diagnostic characters of subspiniform paraterga; lamina lateralis distinctly demarcated from lamina medialis; and lamina medialis long and curved, larger and longer than lamina lateralis.

Based on a comparatively large number of specimens and species of the new genus, our study confirmed that gonopod characters can be used confidently to discriminate the species, just as we found in *Desmoxytes* and *Nagaxytes* (Srisonchai et al. 2018a, 2018b). Using the gonopods in combination with other morphological characters, such as modification of male femora, sternal lobe between male coxae 4, and number of tubercles/cones/spines on collum and on metaterga, further facilitates reliable taxonomic identification. It is particularly interesting that a process on the postfemoral part of the gonopod is found in *S.efefi* sp. n. Only two species of dragon millipede, *Hylomusspecialis* (Nguyen et al. 2005) and *H.spectabilis* (Attems, 1937), have hitherto been known to have this process (z-spine) at the base of the solenophore. However, the overall gonopod characters of *S.efefi* sp. n. are markedly different from the gonopod of the two mentioned *Hylomus* species, warranting its inclusion in the new genus. *Spinaxytes* gen. n., is quite possibly a monophyletic group, considering both gonopodal and non-gonopodal characters. A phylogenetic study using molecular as well as morphological characters seems warranted in order to better understand the taxonomic position and the true relationship of the genus with other dragon millipede genera.

**Figure 30.**
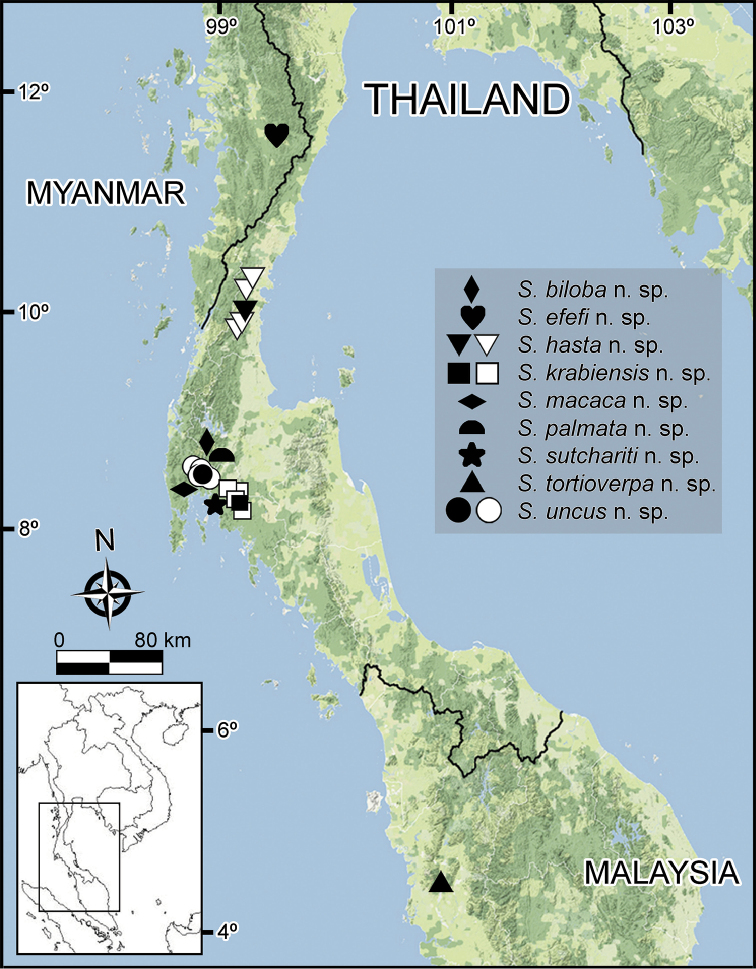
Known distribution of all *Spinaxytes* gen. n. species. Key: black symbols = type locality, white symbols = other localities.

Almost all specimens collected by us were found on humid rock walls in small caves. Therefore, we strongly suspect that all species in this genus prefer to live on rock walls. The black or dark brown body colour makes them difficult to see against dark-coloured rocks. Quite often some specimens of *Desmoxytes* and *Gigaxytes* species are encountered in the same habitat as species of the new genus, but it seems likely that those species live on leaf litter, on the ground or on tree branches instead of rock walls. Considering currently known distributions of species of *Spinaxytes* gen. n., and their restriction to small limestone areas (Figure 30), we regard all described species here as locally endemic. Of the nine species of *Spinaxytes* gen. n., only one (*S.hasta* sp. n.) has been shown to have a somewhat wider range, but it still inhabits less than approximately 50 km^2^ along the coast of Thailand.

The discovery rate of new dragon millipede species has been increasing in recent years (Liu et al. 2014, 2016; Likhitrakarn et al. 2015; Golovatch et al. 2016; Srisonchai et al. 2016, 2018a, 2018b, 2018c). Including the nine new species described here, the diversity of dragon millipedes (*Desmoxytes* + *Hylomus* + *Nagaxytes* + *Gigaxytes* + *Spinaxytes* gen. n.) has now reached 59 species. Dragon millipedes are thus a significant element in the biodiversity of Southeast Asia, especially Thailand and the Malay Peninsula. We believe that the number of endemic dragon millipede species will certainly increase further when collecting efforts in very remote or otherwise difficult-to-access places are made.

## Supplementary Material

XML Treatment for Spinaxytes

XML Treatment for Spinaxytes biloba

XML Treatment for Spinaxytes efefi

XML Treatment for Spinaxytes hasta

XML Treatment for Spinaxytes krabiensis

XML Treatment for Spinaxytes macaca

XML Treatment for Spinaxytes palmata

XML Treatment for Spinaxytes sutchariti

XML Treatment for Spinaxytes tortioverpa

XML Treatment for Spinaxytes uncus
